# Bilayer osteochondral graft in rabbit xenogeneic transplantation model comprising sintered 3D-printed bioceramic and human adipose-derived stem cells laden biohydrogel

**DOI:** 10.1186/s13036-023-00389-x

**Published:** 2023-11-27

**Authors:** Chih-Yun Lee, Swathi Nedunchezian, Sung-Yen Lin, Yu-Feng Su, Che-Wei Wu, Shun-Cheng Wu, Chung-Hwan Chen, Chih-Kuang Wang

**Affiliations:** 1 Ph.D. Program in Life Sciences, College of Life Science, Kaohsiung Medical University, Kaohsiung, 80708 Taiwan; 2 Regenerative Medicine and Cell Therapy Research Center, Kaohsiung Medical University, Kaohsiung, 80708 Taiwan; 3 Orthopaedic Research Center, College of Medicine, Kaohsiung Medical University, Kaohsiung, 80708 Taiwan; 4 Department of Medicinal and Applied Chemistry, College of Life Science, Kaohsiung Medical University, Kaohsiung, 80708 Taiwan; 5 Departments of Orthopaedics, School of Medicine, College of Medicine, Kaohsiung Medical University, Kaohsiung, 80708 Taiwan; 6 Department of Orthopaedics, Kaohsiung Medical University Hospital, Kaohsiung Medical University, Kaohsiung, 80708 Taiwan; 7 Department of Orthopaedics, Kaohsiung Municipal Ta-Tung Hospital, Kaohsiung Medical University, Kaohsiung, 80145 Taiwan; 8 Faculty of Post-Baccalaureate Medicine, College of Medicine, Kaohsiung Medical University, Kaohsiung, 80756 Taiwan; 9 Department of Surgery, Division of Neurosurgery, Kaohsiung Medical University Hospital, Kaohsiung Medical University, Kaohsiung, 80708 Taiwan; 10 Department of Nursing, Asia University, Taichung, 41354 Taiwan; 11 Ph.D. Program in Biomedical Engineering, College of Medicine, Kaohsiung Medical University, Kaohsiung, 80708 Taiwan; 12 Graduate Institute of Medicine, College of Medicine, Kaohsiung Medical University, Kaohsiung, 80708 Taiwan

**Keywords:** Osteochondral, Tissue engineering, Bioceramic scaffold, Digital light processing, Hyaluronic acid methacryloyl, Gelatin methacryloyl

## Abstract

Reconstruction of severe osteochondral defects in articular cartilage and subchondral trabecular bone remains a challenging problem. The well-integrated bilayer osteochondral graft design expects to be guided the chondrogenic and osteogenic differentiation for stem cells and provides a promising solution for osteochondral tissue repair in this study. The subchondral bone scaffold approach is based on the developed finer and denser 3D β-tricalcium phosphate (β-TCP) bioceramic scaffold process, which is made using a digital light processing (DLP) technology and the novel photocurable negative thermo-responsive (NTR) bioceramic slurry. Then, the concave-top disc sintered 3D-printed bioceramic incorporates the human adipose-derived stem cells (hADSCs) laden photo-cured hybrid biohydrogel (HG + 0.5AFnSi) comprised of hyaluronic acid methacryloyl (HAMA), gelatin methacryloyl (GelMA), and 0.5% (w/v) acrylate-functionalized nano-silica (AFnSi) crosslinker. The 3D β-TCP bioceramic compartment is used to provide essential mechanical support for cartilage regeneration in the long term and slow biodegradation. However, the apparent density and compressive strength of the 3D β-TCP bioceramics can be obtained for ~ 94.8% theoretical density and 11.38 ± 1.72 MPa, respectively. In addition, the in vivo results demonstrated that the hADSC + HG + 0.5AFnSi/3D β-TCP of the bilayer osteochondral graft showed a much better osteochondral defect repair outcome in a rabbit model. The other word, the subchondral bone scaffold of 3D β-TCP bioceramic could accelerate the bone formation and integration with the adjacent host cancellous tissue at 12 weeks after surgery. And then, a thicker cartilage layer with a smooth surface and uniformly aligned chondrocytes were observed by providing enough steady mechanical support of the 3D β-TCP bioceramic scaffold.

**Supplementary Information:**

The online version contains supplementary material available at 10.1186/s13036-023-00389-x.

## Introduction

Cartilage tissues are highly specialized, well hydrated, and possess low friction, wear-resistant characteristics, which ultimately help in easy joint movement [1]. Regrettably, cartilage loses its usual structure and function with aging or due to some traumas or injuries. And due to its cellular nature, cartilage also fails to produce any significant healing response, eventually leading to long-term complications [2]. Current treatments for cartilage injury often involve surgical interventions to remove affected tissues and insert transplanted osteochondral plugs as a replacement [3, 4]. The most common methods include 1) arthroscopic drilling to get bone marrow for cartilage repair; 2) mosaicplasty to replace both cartilage and subchondral bone with an osteochondral graft; 3) autologous cell transplantation to inject pre-expanded cells under an autologous periosteal flap sutured on the cartilage; and 4) prosthetic replacement to remove the joint and replace it with a mechanical device, While these procedures may provide relief from pain and restore joint mobility, they can present long-term complications [3, 5].

Tissue engineering strategies, integrating the elements of cells, scaffolds, and bioactive factors, provide a potential solution for repairing and regenerating native osteochondral tissues [6, 7]. Although cell transplantation-based tissue engineering treatment for human cartilage repair was introduced almost two decades ago, current cartilage tissue engineering strategies cannot yet fabricate new perfect cartilage tissue, that is indistinguishable from native cartilage concerning the zonal organization, extracellular matrix (ECM) composition, and mechanical properties [8]. Based on the marked difference in the composition and properties of the cartilage layer and subchondral bone, the composite scaffolds strategies containing a chondrogenic and an osteogenic compartment have been recently developed to mimic the native osteochondral tissue structurally and functionally [9–12]. Some approach involves independent fabrication or culture of chondrogenic and osteogenic layers and a subsequent joining of the two layers by suturing or gluing [10]. Among them, Cao et al*.* [11] reported that fibrin glue containing chondrocytes was able to coagulate on a precultured polycaprolactone (PCL) scaffold containing osteoblasts, to form a composite, and the scanning electron microscopy observed the co-cultured constructs revealed cell proliferation and matrix production within each compartment, as well as matrix integration at the interface. However, in a similar study by Lima et al*.* [12], the incorporation of devitalized bone under chondrocyte-seeded agarose was found to reduce the biochemical and mechanical properties of the chondrogenic layer, although good integration between layers was observed. In addition, Lee et al*.* employed human adipose-derived stem cells (hADSCs) and nanofibers coated with either transforming growth factor-β3 and bone morphogenetic growth factor-2 to create two types of composite spheroids for chondrogenesis or osteogenesis [13]. From these approaches, this osteochondral composite technique is advantageous because it can achieve spatial control of cell types and bioactive factor release in a minimally invasively [14].

Despite promising results from the in vivo studies above the osteochondral composite technique, challenges remain in constructing a well-integrated bilayer scaffold that should be guided by the chondrogenic and osteogenic differentiation of cells in different regions of the composite scaffold. Such as subchondral bone can obtain the cell source from bone marrow-derived mesenchymal stem cells (BMSCs), then the chondral layer repair can be from the human adipose-derived stem cells (hADSCs) of another site. In addition, autologous transplantation requires two surgeries, and the cell source is still limited. Rabbits are phylogenetically and anatomically more closely related to humans than rodents [15]. However, previous reports that mesenchymal stem cells (MSCs) derived from different adult tissues, such as bone marrow, adipose, umbilical cord, and liver, have advantages for the immunosuppressive effect [16, 17]. Thus, this study investigates the possibility of creating a xenogeneic human adipose-derived stem cell-laden biohydrogel for chondral layer repair in the rabbit model. Fortunately, we have developed a novel photo-cured hybrid biohydrogel system (HG + 0.5AFnSi) comprised of hyaluronic acid methacryloyl (HAMA), gelatin methacryloyl (GelMA), and 0.5% (w/v) acrylate-functionalized nano-silica (AFnSi) crosslinker recently, which concluded that provide a suitable environment for chondrogenesis of hADSCs [18].

**Fig. 1:**
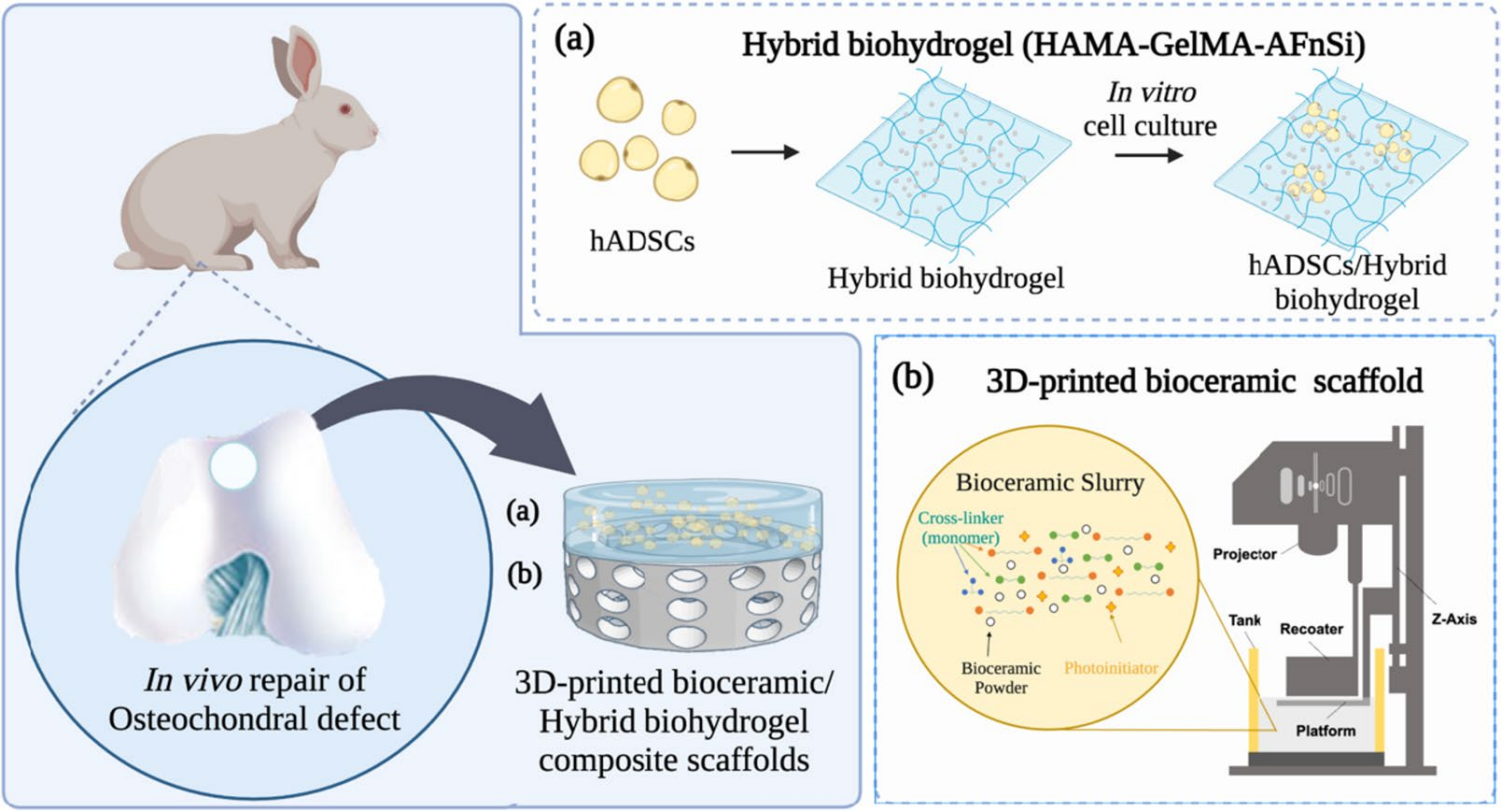
A schematic diagram of the well-integrated bilayer osteochondral scaffold is expected to guide stem cells' chondrogenic and osteogenic differentiation and provide a promising solution for osteochondral tissue repair. The human adipose-derived stem cell (hADSCs) laden photocured hybrid biohydrogel comprised of hyaluronic acid methacryloyl (HAMA)/gelatin methacryloyl (GelMA) (1/1) and 0.5% (w/v) acrylate-functionalized nano-silica (AFnSi) crosslinker, which provide a suitable environment for cartilage regeneration. **a** The concave-top disc structure of the subchondral scaffold with the denser intermediate layer and the well-open interpenetrating pore channels structure of the 3D-printed bioceramic scaffold is made using digital light processing (DLP) technology and the novel photocurable negative thermo-responsive (NTR) bioceramic slurry. **b**

Therefore, we want to further develop a biomimetic osteochondral integration scaffold from top to bottom and mimic the bilayer tissue of cartilage. The well-integrated subchondral bone scaffold includes the subchondral intermediate layer (cortical plate) and the subchondral well open interpenetrating pore channels (trabecular bone) structure to reduce the interference of BMSCs to the upper chondral layer of hADSCs. In addition, the subchondral scaffold approaches based on the newly developed denser 3D-printed bioceramic scaffold process, made using a digital light processing (DLP) technology and through the vacuum mixing process for novel photocurable negative thermo-responsive (NTR) bioceramic slurry. Then, the 3D sintered bioceramic scaffold could have dense and well open interpenetrating pore channels after 1150°C sintering under silicon oil. The rationale for the NTR bioceramic slurry system is that the intramolecular hydrogen bond of the poly(N‐isopropylacrylamide) (pNIPPAm) hydrogel shrinks since the temperature is above 32–33°C. Then it can shrink drainage evenly of the slurry to pack ceramic powders closely under silicon oil enveloped [19–22]. However, beta-tricalcium phosphate (β-TCP) has an osteoconductive and biodegradation bioceramic compartment at above 1150°C sintered, which is used to provide essential mechanical support and promote bone formation for cartilage regeneration in the long term [22–25]. Besides discussing the successful printing parameters of different bioceramic slurry composition ratios on the top-down DLP machine, it also analyzes the bioceramic sintered body's physical/chemical properties and cell viability. In short, we developed the biomimetic bilayer osteochondral graft would be a method based on the hypothesis that a stable mechanical subchondral bioceramic of the sintered 3D β-TCP scaffold would enhance bone growth for BMSCs and support cartilage regeneration in the long term, the chondral compartment was created from the xenogeneic transplantation of hADSCs laden photo-cured hybrid biohydrogel (HG + 0.5AFnSi) system for cost‐effective cartilage regeneration, shown in Fig. 1.

## Materials and methods

### Materials

The raw materials for the 3D-printed bioceramic scaffold fabrication are as followed, the 70% β-TCP powder purchased from Sigma-Aldrich Inc. (USA) and combined with a 30% amount of hydroxyapatite (HAp) powder was purchased from CAPTALS®, Plasma Biotal Limited (UK). Other photo-cured bioceramic slurry components, such as the organic solution of ethanol and the trimethylolpropane triacrylate (TMPTA), were purchased from Sigma-Aldrich Inc. (USA). The negative thermal responsive monomer of n-isopropyl-acrylamide (NIPPAm), photo-initiator (P.I.) of phenyl-bis (2,4,6-trimethyl-benzoyl) phosphine oxide Irgacure®819 (I819) were purchased from Tokyo chemical industry CO., LTD (Japan). The poly(ethylene glycol) diacrylate (*PEGDA*) of 700 Da was purchased from a local supplier of VIEW STAR CO., LTD (Taiwan). These chemicals were used without further purification.

The raw materials of the photo-cured hybrid biohydrogel system (HG + 0.5AFnSi) were according to the practice in our previous literature and briefly introduced as follows [18]: the hyaluronic acid (molecular weight of 2000 kDa) was purchased from Kikkoman (FCH-200, Japan), gelatin from porcine skin (Type B), and methacrylate anhydride (MA) (molecular weight of 154.16 Da) were purchased from Sigma-Aldrich (USA). The P.I. of lithium phenyl-2,4,6-trimethyl-benzoyl phosphinate (LAP), nano-silica (SiO_2_; nSi), 3-acryloxypropyl trimethoxysilane (APMS) were also obtained from Sigma-Aldrich (USA). All other solvents were purchased from Merck (Germany), TEDIA (Fairfield, USA), or J. T. Baker (Phillipsburg, USA). These chemicals were all analytical/reagent grade and were used without further purification.

### Fabrication of 3D-printed bioceramic scaffolds

#### Preparation of bioceramic slurry

The following steps were used to create the photo-cured bioceramic slurry with the negative thermal responsive monomer of NIPPAm. Initially, to test these optimal crosslink ratios of NIPPAm, PEGDA, TMPTA, and ethanol solvent with a solids content of 30 vol% of β-TCP/HAp (70/30 weight ratio) powders to determine the printable parameters by DLP process. After the resin ratio was determined, the 2.6 wt% I819 of P.I. was added and mixed for 1 h under the dark on a stirrer (LS-2R, Mandarin Scientific Co., Ltd., New Taipei City, Taiwan) at a speed of 500 revolutions/min (rpm). Second, three kinds of solid phase volume percent (30 vol%, 35 vol%, and 40 vol%) at the fixed portions of β-TCP and HAp powders in 70:30 in a weight ratio (%) were vigorously agitated for 2 h under the dark environment. i.e. the agitator was covered with aluminum foil to avoid light at room temperature (~ 25 ℃).

#### Rheological test of bioceramic slurry

Literature indicated that preparing low-viscosity ceramic slurry is the key limitation for successfully printing the 3D ceramic green-body under DLP printer technology [26–28]. However, these viscosities of slurries with different bioceramic powder volume loadings were evaluated at room temperature. Each bioceramic slurry (*n* = 6) was measured at shear rates from 0 s^−1^ to 200 s^−1^ using a rheometer (TA Instruments Discovery HR 20, New Castle, DE, USA) in planar geometry (d = 25 mm).

#### Design and fabrication of 3D-printed bioceramic scaffolds for osteochondral use

**Fig. 2:**
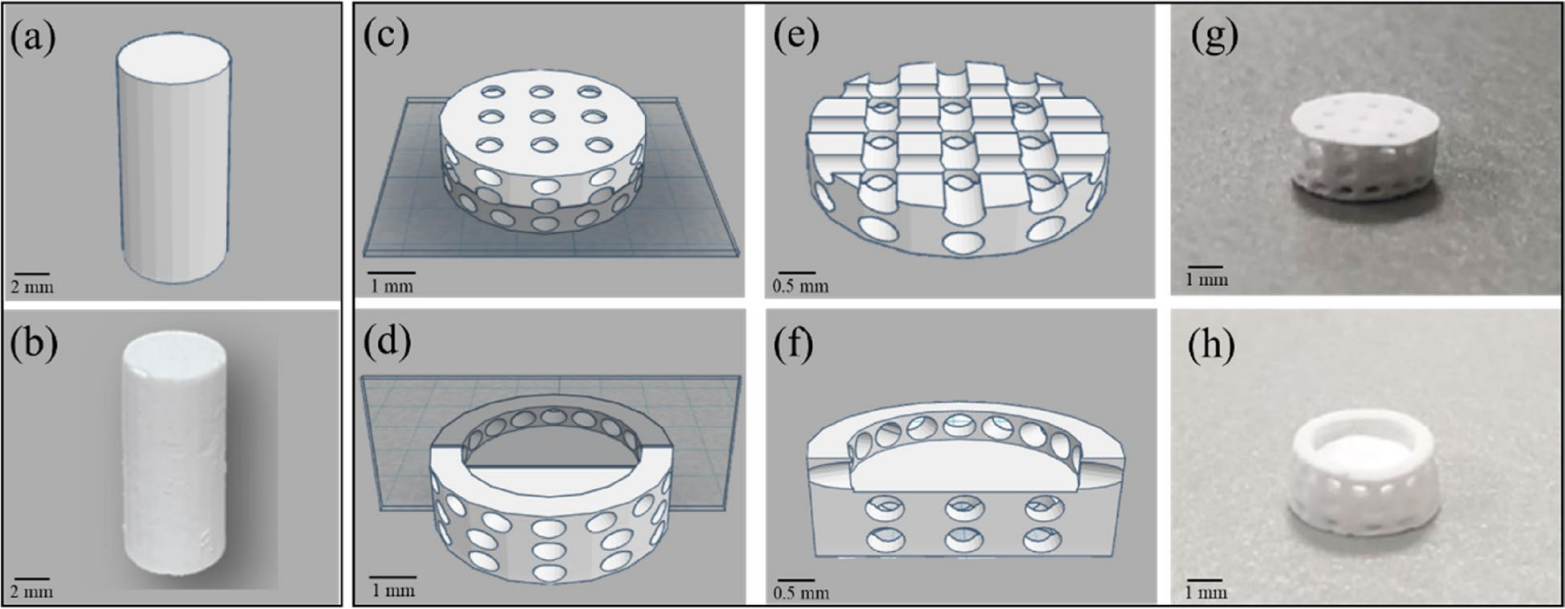
The STL images of two geometries of the 3D-printed bioceramic model were designed as follows: The cylindrical compression sample (**a**), the concave-topped disk structures views of the bottom (**c**), and the top (**d**). The cross-section views of concave-top disk structures also showed the STL image of a horizontal section (**e**) and a vertical section (**f**). Furthermore, the two kinds of 3D-printed sintered bioceramic images were obtained. The 3D cylinder bioceramic sample (**b**), the bottom view (**g**), and the top view (h) of the concave-top disc structure of the 3D-printed bioceramic scaffold

Two geometries of 3D-printed bioceramics were designed in this study. One of them is a cylinder mode with a diameter of 4.8 mm and a height of 12.0 mm, as shown in Fig. 2a, for compressive strength analysis. The second type is a concave-top disc structure of subchondral scaffold with the denser intermediate layer and the well open interpenetrating pore channels structure used for osteochondral defect regeneration study in rabbit animal experiments (Fig. 2 c, d). The outer diameter of the concave-top disc is 4.0 mm, and the height is 1.8 mm. In addition, the inner disk structure is designed with multiple open interpenetrating pore channels with a diameter of 0.5 mm. It is widely recognized in the literature that the optimal pore size for bone graft materials to support the growth of bone cells falls within the range of 100–1000 μm [29] However, the denser intermediate layer serves as the cortical plate can be prevented the BMSCs in the subchondral bone region from migrating to the chondral layer and creating interference, which will be convenient for studying the cartilage regeneration effect of the hADSCs laden with novel photo-cured hybrid biohydrogel (HG + 0.5AFnSi) system on osteochondral scaffold systems. Therefore, the open interpenetrating pore channels are designed not to pass through the top of the concave roof, as shown in the longitudinal and cross-sectional models in Fig. 2 e, f. In our design of the concave structure within the 3D bioceramic scaffold, we also incorporated pore structures on the top side. The purpose of these side pores was to enhance the adhesion between the photo-cured biohydrogel and the 3D bioceramic scaffold.

Both 3D-printed bioceramic scaffolds were fabricated using a top-down DLP 3D printer (Octave Light R1, Octave Light Ltd., Hong Kong), we conducted cross-testing analyses to assess various printing conditions and selected a set of optimal parameters, with the main parameters controlled as follows: an exposure time of 5 s at a UV wavelength of 405 nm, and each layer thickness set to 50 μm. However, a standard stereolithography (STL) file was exported from a 3D CAD model of the scaffold created online using the software Tinkercad® from Autodesk® (Autodesk Inc., CA, USA) in this study. Then, the STL file was imported into the DLP printer of Octave Light R1 and set the appropriate printing parameters to create the green-body of the 3D-printed bioceramic. In addition, the as-received 3D-printed bioceramic green-body still needs to remove their uncured paste slurry before sintering processing from the water with the ultrasonic cleaner and immerse in water for 10 min in order to replace ethanol to water for pNIPPAm shrinking upon heating. In addition, silicone oil (polydimethylsiloxane purchased from Sigma-Aldrich Inc., USA) was used to seal the surface of the as-received 3D-bioceramic green-body, which can promote the uniform drainage of water, causing these ceramic powders to hold together tightly. Therefore, the as-received 3D-printed bioceramic green-body cover silicon oil and dried in an oven at 60 ℃ for 1 h. Finally, the dried 3D-printed bioceramic green-body after stable shrinkage, has to undergo a debinding at 500°C and sintered at 1150°C for 2 h to obtain a densified sintered 3D bioceramic scaffold in the muffle furnace (LH 15/14, Nabertherm, Lilienthal, Germany). The final 3D cylinder sintered bioceramic samples and the concave-top disc sintered 3D-printed bioceramic scaffolds were obtained and shown in Fig. 2b, g-h.

### Characterization of as-received 3D-printed bioceramic green-body and sintered scaffolds

#### Thermogravimetric analysis

Thermogravimetric analysis (TGA) is a technique in which the mass loss of a specimen in a controlled atmosphere is measured as the time or temperature changes. In this study, the stable shrinkage and dried the as-received 3D-printed bioceramic green-body specimen at the 60°C oven, which was utilized for the subsequent thermal debinding/degradation temperature experiments to determine the ideal sintering parameters. Thermogravimetric analysis (TGA) (Q500, TA Instruments, USA) was performed in an air atmosphere heated from 25°C to 800°C at 10°C/min.

#### X-ray diffraction analysis

The phase structure of as-received HAp powders, β-TCP powders, and the sintered 3D- bioceramic scaffolds were analyzed for the 2θ angular range from 20° to 40° using X-ray diffraction (XRD; TTRAX III; Rigaku, Japan).

#### Surface morphology and element analysis of 3D-printed bioceramic scaffold

The morphology and open interpenetrating pore channels structure from the top and lateral surface of sintered 3D-printed bioceramic scaffolds were observed using field emission scanning electron microscopy (SEM, Hitachi SU8000, Tokyo, Japan). These SEM samples were dehydrated first and coated with a thin layer of conductive gold. The elemental composition percentage and the Ca/P atomic ratio of sintered 3D-printed bioceramic scaffolds were also investigated by SEM fitted with energy-dispersive X-ray spectroscopy (SEM–EDS) at an accelerating voltage of 10.0 kV.

#### Porosity evaluation

The apparent density and apparent porosity of the sintered 3D-printed ceramic samples were tested by the Archimedes method using an electronic densitometer (GP-120C, Matsuhaku, Taiwan). Brief experiment processes were as follows: The dry weights of the sintered 3D-printed ceramic samples (m_1_) were recorded. Then, these sintered 3D-printed ceramic samples were immersed in distilled water until no bubbles appeared in the beaker, and the submerged mass (m_2_) was measured. However, the apparent solid volume (V_as_ = m_1_-m_2_) includes sintered 3D-printed ceramic and closed pores [30]. The apparent solid density is the mass divided by the apparent solid volume as described below:

1 $$\mathrm A\mathrm p\mathrm p\mathrm a\mathrm r\mathrm e\mathrm n\mathrm t\,\mathrm s\mathrm o\mathrm l\mathrm i\mathrm d\,\mathrm d\mathrm e\mathrm n\mathrm s\mathrm i\mathrm t\mathrm y\left({\mathrm D}_{\mathrm{AS}}\right)={\mathrm m}_1/{\mathrm V}_{\mathrm{as}}$$

Measure the bulk density requires the dimension values of the concave-top disc sintered 3D-printed bioceramic scaffold and calculating the bulk volume (VB). The VB is the outer volume of concave-top disc sintered 3D-printed bioceramic scaffolds, which includes the real volume of sintered 3D-printed bioceramic objects, open interpenetrating pore channels, open pores, and closed pores. However, a digital vernier caliper (CD-6 “CS, Mitutoyo, Japan) and a digital microscope (Microdirect 1080P, Celestron, USA) were used to measure the dimensions of sintered 3D-printed bioceramic scaffolds, including the outer/inner diameter and depth of the concave-top disc, and the height of the disc. The following equation then calculated the bulk density:

2 $$\mathrm B\mathrm u\mathrm l\mathrm k\,\mathrm d\mathrm e\mathrm n\mathrm s\mathrm i\mathrm t\mathrm y\left({\mathrm D}_{\mathrm B}\right)={\mathrm m}_1/{\mathrm V}_{\mathrm B}$$

The total porosity (%), given by the sum of the closed pores, open pores, and open interpenetrating channels, was calculated according to

3 $$\mathrm{Total}\ \mathrm{porosity}\left(\%\right)=\left(1\frac{\mathrm{m}_{1}}{\mathrm{pxV}_\mathrm{B}}\right)\times100$$

where ρ is the theoretical density of the sintered 3D-printed bioceramic scaffold from the ratio of β-TCP [22, 31]. Regarding the schematic diagram of apparent density, bulk density, and porosity, please refer to the sFig 1 of the supplemental information. Three specimens for each group were tested to calculate the average apparent density, bulk density, and porosity. The results are expressed as the mean ± the standard deviation (SD).

#### Compressive strength measurement

The specimens were tested for compressive strength using a universal testing machine (3365 MACHINE, Instron®, USA) with a load cell of 10 kN. The loading speed was set at 0.5 mm/min. At least six specimens from each group were tested to obtain the average mechanical strength to ensure statistical significance. Engineering stress–strain curves were calculated by normalizing the captured load vs. displacement data using each sample's initial external dimensions (ϕ 4.8 × height 12.0 mm). The results are expressed as the mean ± standard deviation (SD).

#### Cytotoxicity assay

Cytotoxicity assay procedures are based on ISO 10993–5 medical devices and cell culture methods using extraction of test substances. The brief procedure is described below: the DMEM (Dulbecco's Modified Eagle Medium) containing 10% fetal bovine serum and 100 U/mL penicillin/streptomycin was used as the extraction buffer. These test examples were sintered 3D-printed bioceramic scaffolds autoclaved at 121 ℃ for 15 min before extraction. The ratio of the test group sample to extractant was 0.2 g/mL in DMEM, and the extraction solution was conducted at 37 ± 1 ℃ for 72 ± 2 h. And then, the group consisting entirely of cells was used as a control, the group containing alumina (Al_2_O_3_) was used as a negative control, and the 10% sodium dodecyl sulfate (SDS) was used as a positive control. However, the normal control, positive control, and negative control groups were treated under the same extraction conditions.

The 3-(4,5-dimethylthiazol-2-yl)-2,5-diphenyltetrazolium (MTT) assay is an assay that detects the concentration of mitochondrial dehydrogenase, which represents an indicator of cell viability, proliferation, and cytotoxicity. Then, the NIH-3T3 cells were cultured, and the cytotoxicity of the sintered 3D-printed bioceramic scaffolds were evaluated using the MTT assay (purchased from Sigma, USA) in DMEM. Firstly, the NIH-3T3 cells were seeded in 96-well plates at a density of 1 × 10^4^ cells per well and incubated at 5% CO_2_ for 24 h at 37 ℃ to reach 80% confluence. After 24 h, these cell cultures were replaced with extracted DMEM from the sintered 3D-printed bioceramic and incubated at 37 ℃ for 24 and 48 h for MTT assays. For the MTT evaluation, 50 μL of MTT reagent in phosphate-buffered saline (PBS) was added to each well and incubated for 2 h at 37 ℃ in the dark. After 2 h of incubation, the supernatant was aspirated, and the formazan crystal was dissolved in 100 μL of DMSO. The light absorbance was measured at 595 nm using an ELISA microplate reader. Cell survival and viability were expressed as a percentage of the absorbance of the NIH-3T3 cells. In addition, the MTT assays were repeated in six independent experiments. All the data were statistically analyzed to express the mean ± SD (*n* = 6). The results of the t-test method were compared to the control group or between treatment groups, with *p < 0.05 and **p < 0.01 being used as significance levels.

### Preparation and characterization of HAMA-GelMA (HG) base hybrid biohydrogel

To evaluate the cartilage regeneration on an osteochondral model of rabbit, the hADSCs will be specified to add into the novel photocurable hybrid biohydrogel system, which consists of the 1:1 HAMA-GelMA (HG) hybrid biohydrogel with 0.5% (w/v) acrylate-functionalized nano-silica (AFnSi) crosslinker (HG + 0.5AFnSi). Whatever the HG hybrid biohydrogel and AFnSi were performed following previously reported protocols, the relevant physical/chemical characteristics were consistent, and their evidence has been described in our previous paper [18]. A brief conclusion is that the photo-cured hybrid biohydrogel of HG + 0.5AFnSi that have the optimum conditions to support the hADSCs survival and differentiation of chondrogenesis. For example, the pore size was near 75.0 ± 0.2 μm, the swelling ratio was around 35%, the compressive modulus was up to 35.0 ± 1.5 kPa, and the in vitro degradation rate is approximately 25% after 30 days. However, the HG + 0.5% (w/v) AFnSi had considerably more glycosaminoglycans (GAGs) deposition and cartilage marker gene expression [18]. Therefore, the greatest option for cartilage tissue regeneration would be this bioinspiration hybrid biohydrogel of HG + 0.5AFnSi.

### Animal experiments

#### Isolation and culturing of human adipose-derived stem cells

This study examined the isolation of hADSCs from human subcutaneous adipose tissue, which was performed according to a previously described procedure [18]. The hADSCs were isolated from subcutaneous adipose tissue of human patients during orthopedic surgery after obtaining informed consent from all patients and approval from the Kaohsiung Medical University Hospital ethics committee (KMUH-IRB-E(II)-20150193). Briefly, 3.0 g of human subcutaneous adipose tissue was extracted and cut into small pieces using scissors. The minced tissues were digested with 1.0 mg/mL of type I collagenase at 37 °C under 5% CO_2_ for 24 h. Then, centrifugation was performed at 1000 rpm for 5 min. The pellet was collected and washed twice with PBS. After that, the pellet was resuspended in K-NAC medium, and the cells were counted and plated in a 100 mm culture dish. Subsequently, the hADSCs attached to the culture plate were maintained at 37 °C in a 5% CO_2_ incubator. The K-NAC medium used in this study is suitable for the isolation and expansion of hADSCs described in the preceding study. The K-NAC medium mainly contains Keratinocytes-SFM (Gibco BRL, Rockville, MD) supplemented with 25.0 mg of bovine pituitary extract (BPE), 2.5 µg of human recombinant epidermal growth factor, 2.0 mM N-acetyl-l-cysteine, 0.2 mM L-ascorbic acid, and 5% FBS. The first medium change was performed after 24 h, and the hADSCs unadhered to the plate were washed off using PBS. Subsequently, the fresh medium was changed every two days. The cells grew to nearly 90% confluence and were subcultured for further cell studies [18].

#### Preparation of bilayer osteochondral graft

**Fig. 3:**
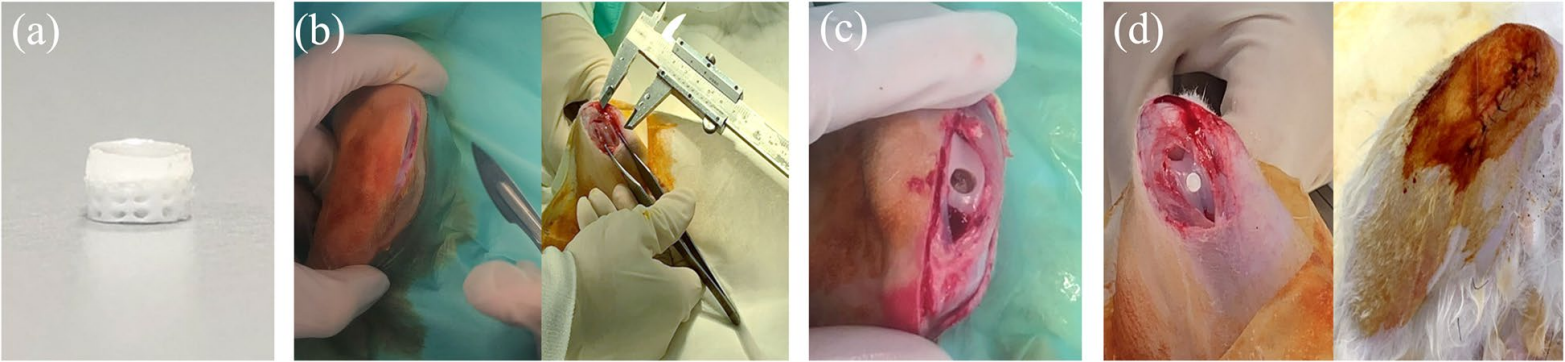
An osteochondral defect was generated in the rabbit knee patellofemoral groove surface. The photo of the xenogeneic tissue-engineered bilayer osteochondral graft (**a**). The steps involved in the implantation procedure are as follows: the Vernier caliper was used to measure the perforation position after bone opening (**b**), creating an osteochondral defect 4.0 mm in diameter and 3.5 mm in height (**c**). The bilayer osteochondral graft comprising the 3D-printed bioceramic scaffold with/without human adipose-derived stem cells laden with biohydrogel was implanted in rabbits (**d**)

The efficacy of the biomimetic osteochondral integration bilayer scaffold is the most practical solution for clinical application. For this study, the osteochondral graft made by a newly developed densified concave-top disc sintered 3D-printed bioceramic scaffold incorporates three kinds of photo-cured biohydrogel system, which will contain the 2×10^4^ hADSCs or not. However, these biohydrogel systems will be HAMA, HG, and HG + 0.5 wt% AFnSi in the basal medium. The biomimetic osteochondral integration bilayer scaffold procedure is briefly described as follows: First, the concave-top disc sintered 3D-printed bioceramic and the biohydrogel solution to be implanted were sterilized by autoclaves or UV light overnight, and then the culture medium and photoinitiator solution were filtered with a 0.25 μm filter for later use. In the laminar flow sterile cabinet, the photo-cured biohydrogel with or without 2 × 10^4^ hADSCs were incorporated into the top layer of 3D-printed bioceramic scaffolds. To create the cross-linking of the interface between the biohydrogel and the porous 3D-printed bioceramic scaffold required the employment of a UV 365 nm photo-curing method for 120 s. Then, the bilayer osteochondral graft comprising a 3D-printed bioceramic scaffold with or without hADSCs loaded with hybrid biohydrogel was used for the osteochondral defect of the rabbit model. The optical photo of the tissue-engineered bilayer osteochondral graft is shown in Fig. 3a. We have previously provided a detailed in vitro analysis of the ability of our biohydrogel system to promote chondrogenic differentiation of ADSCs in our published paper [32]. However, the ADSCs within this biohydrogel can exchange nutrients and metabolites through diffusion in the external physiological environment. The physiological effects of external compression would enhance this process, but achieving this requires not only the viscoelastic properties of the biohydrogel but also a slower degradation rate. In this study, we did not conduct additional in vitro degradation experiments for this bilayer osteochondral graft. This reason is based on our previous research on the in vitro degradation of our 3D bioceramic scaffold, which showed minimal degradation over a 4-week immersion period [33] and even an increase in weight due to the precipitation of hydroxyapatite in the physiological environment. Therefore, conducting animal experiments directly provides a more representative assessment of the actual degradation rate.

#### Surgical procedure for osteochondral regeneration in rabbit

The animal study was approved (No: 106217) by the Ethical Committee of the Kaohsiung Medical University (KMU) Animal Research Center in Taiwan. However, all New Zealand white rabbits were also housed at KMU Animal Research Center, and experiments were conducted in accordance with the guidelines, and adequate measures were taken to minimize the pain and discomfort of the animals. The preoperative preparation and surgical procedures are briefly described below: three-month-old New Zealand rabbits (weighing 3.1 to 3.8 kg) were used to assess the efficiency of osteochondral regeneration. We established one osteochondral defect model in the center of the patellar (trochlear) groove of the rabbits. The brief operation process of the osteochondral defect and implanted the bilayer osteochondral graft was shown in Fig. 3 b-d.

In the first-phase in vivo study, the osteochondral repair groups were divided into five groups for 8 weeks evaluation, which included the blank group (osteochondral defect only), the bilayer osteochondral grafts comprising of (1) 3D-printed bioceramic scaffold incorporated the photo-cured hybrid biohydrogel of HG group (HG/3D β-TCP), (2) 3D-printed bioceramic scaffold incorporated the photo-cured hybrid biohydrogel of HG + 0.5% AFnSi group (HG + 0.5AFnSi/3D β-TCP), (3) 3D-printed bioceramic scaffold incorporated the hADSCs laden with photo-cured hybrid biohydrogel of HG group (hADSC + HG/3D β-TCP), and (4) 3D-printed bioceramic scaffold incorporated the hADSCs laden with photo-cured hybrid biohydrogel of HG + 0.5% AFnSi group (hADSC + HG + 0.5AFnSi/3D β-TCP). In the second-phase in vivo study, the osteochondral repair groups just were divided into three groups for 12 weeks of evaluation, which included the blank group, 3D-printed bioceramic scaffold incorporated the hADSCs laden with the photo-cured biohydrogel of hADSC + HAMA/3D β-TCP group, and the hADSC + HG + 0.5AFnSi/3D β-TCP group.

Whatever, the anesthesia was administered by intraperitoneal injection of ketamine (Ketalar®, Parke-Davis, Taiwan) in conjunction with xylazine-hydrochloride (Rompun®, Bayer HealthCare, Germany) injection through the intraarticularly of the rabbit with a dose of 1.0 mL Kg^−1^. The rabbit was secured in position, and the surface of the knee joint was exposed via skin and muscle incisions. With the effusion of bone marrow blood in the center of the patella groove, a hand drill was utilized to generate an osteochondral defect 4.0 ± 0.1 mm in diameter and 3.5 ± 0.1 mm in height. Each scaffold was transplanted into one defect. After placing the implant of the bilayer osteochondral graft, the synovium, capsule, and skin of the knee joint were each sutured separately. After they had fully recovered from the effects of the anesthetic, each of the rabbits in the study quickly began standing on all four legs. All rabbits received intramuscular injections of antibiotics for three days. After surgery, the rabbits were housed individually in cages with free access to food and water. 8 and 12 weeks after surgery, the rabbits were sacrificed, and cartilage tissue was harvested. The results of the animal experiments further validate the effectiveness of this design, demonstrating that there is no slippage between the two components.

#### Soft X-ray radiograph analysis

Eight or twelve weeks after surgical implantation of the bilayer osteochondral grafts were the time points for assessing osteochondral defect regeneration. To ensure defects after surgery and before sacrifice, the rabbits were put through live X-ray radiological exam using an X-ray machine (SOFTEX, M-100, Japan) at 70 kVp, 2 mA, and a 44-cm film-radiation beam distance for a 1.5-s exposure time [17, 34].

#### Hematoxylin and eosin (HE) stain, Safranin-O green stain, and Masson’s trichrome stain for histomorphometric analysis

The knee joint is opened, and a preliminary visual inspection assesses the neocartilage. The bilayer osteochondral graft in the rabbit’s knee joint tissue specimens were harvested and fixed in 4% paraformaldehyde at 4 °C for 24 h. Eight and twelve weeks after surgical implantation, the bilayer osteochondral grafts in the rabbit knee joint tissue specimens were harvested and evaluated for histomorphology. All knee joint tissue specimens were decalcified, fixed, dehydrated, and paraffin-embedded, as described previously [17, 34].

The 5-μm thick knee joint tissue specimen sections were removed and stained with hematoxylin and eosin (HE stain; Santa Cruz, Santa Cruz, CA, USA). A microscope with 40 × and 400 × magnification to observe, equipped with a digital CCD camera (Eclipse 50i; Nikon Inc., Michigan, USA), provided images of the sections. The repair area around the osteochondral defect was measured by using Image-Pro Plus 5.0 software (Media Cybernetics Inc., USA). Safranin-O and fast green staining were used to evaluate the GAGs production in the chondral layer of bilayer osteochondral xenograft constructs in vivo [17]. To evaluate GAGs production, the 5-μm thick sections of the knee joint tissue specimen sections harvested from rabbit knee were stained with 1% Safranin-O and counterstained with 1% fast green (Sigma, Saint Louis, MO, USA). Sections were then counterstained with 0.75% hematoxylin. Masson's trichrome staining was used to examine the collagen accumulation and orientation in general. In brief [33], the 5-μm thick sections of the knee joint tissue specimen sections placed on standard microscopy slides. After deparaffinization and rehydration, the slides were immersed in Bouin’s solution (HT 10132, Sigma-Aldrich) at 56˚C for 15 min. Subsequently, the slides were washed with tap water for 5 min. Next, the sections were stained in Weigert’s hematoxylin for 5 min, washed with tap water for 5 min, and rinsed in distilled water. Next, the slides were stained in Biebrich scarlet-acid fuchsin for 5 min, rinsed in distilled water, incubated in phosphotungstic-phosphomolybdic acid for 5 min, dyed with aniline blue for 5 min, and fixed in 1% acetic acid for 2 min. Finally, the slides were rinsed with distilled water, dehydrated, and mounted. The bright-field microscope was used to acquire the histological images.

## Results and discussion

We have previously developed the effectiveness of osteoconductive scaffolds with bicontinuous phases (*i.e.* channel phase and bioceramic phase) of 3D-printed bioceramic based on reverse negative thermo-responsive hydrogels (NTRH; poly(*N*-isopropylacrylamide)-*co-*(methacrylic acid)) bioceramic slurry and robotic deposition additive manufacturing (RDAM) [22]. The RDAM still has the following key disadvantages [22, 35–37]: (1) RDAM has a poor surface finish and cannot produce fine features structure. Because the hardness of ceramic slurry is insufficient, the RDAM will be more difficult to produce a pore structure in the printing x/y-axis direction. (2) Due to the poor interlayer bonding strength common and the NTRH molecular alignment directionality of ceramic slurry. Therefore, the RDAM has a high level of anisotropy in the XYZ direction leading to uneven sintering shrinkage and even cracks in larger samples.

Therefore, this study developed the DLP fabrication incorporated with the novel photocurable NTR bioceramic slurry. In addition to solving the above problems, this DLP is suitable for providing well-integrated subchondral bone scaffold approaches based on the exquisite and dense 3D-printed bioceramic skeleton process. However, the specimen of DLP shall be more isotropic since the green-body shrinkage evenly. The ingredient formulation of the novel photocurable NTR bioceramic slurry was developed based on the following rationale in this study: (1) Select some of the water-soluble monomeric cross-linking agents so that they can successfully 3D print stereoscopic structure specimens on a DLP machine. (2) The NTR monomer proportion of NIPPAm is as high as possible to achieve a better drainage shrinkage effect. (3) Because the HAp will decompose to β-TCP during 1000°C sintering, it can make the volume little increase can enhance the mechanical properties of the β-TCP sintered body that results [22, 38]. The sintered near pure β-TCP bioceramics can be obtained following the initial β-TCP/HAp mole ratio of 70/30 raw materials based on our previous experiments [22]. However, these weight ratios of 90.0/6.4/5.1/100.0 suitable ingredients of NIPPAm, PEGDA, TMPTA, and ethanol have been evaluated and confirmed at 30 vol% solids in β-TCP/HAp (70/30 molar ratio) powder because they can have obtained successful printable parameters under the DLP process system. The P.I. was also determined by 2.6 wt% I819 in this study. In addition, the feasibility confirmation of ingredient design, the NTR bioceramic slurry to form a 3D structure using the DLP process still needs consideration of other parameters comprising slurry viscosity (or flowability), ceramic solid content, exposure energy, curing depth, etc.… Due to these factors, final product characteristics will be evaluated to confirm the capabilities of photo-cured 3D structures and whether they will actually be successful.

### Formulation and viscosity of optimal NTR bioceramic slurry for DLP 3D printing

**Fig. 4:**
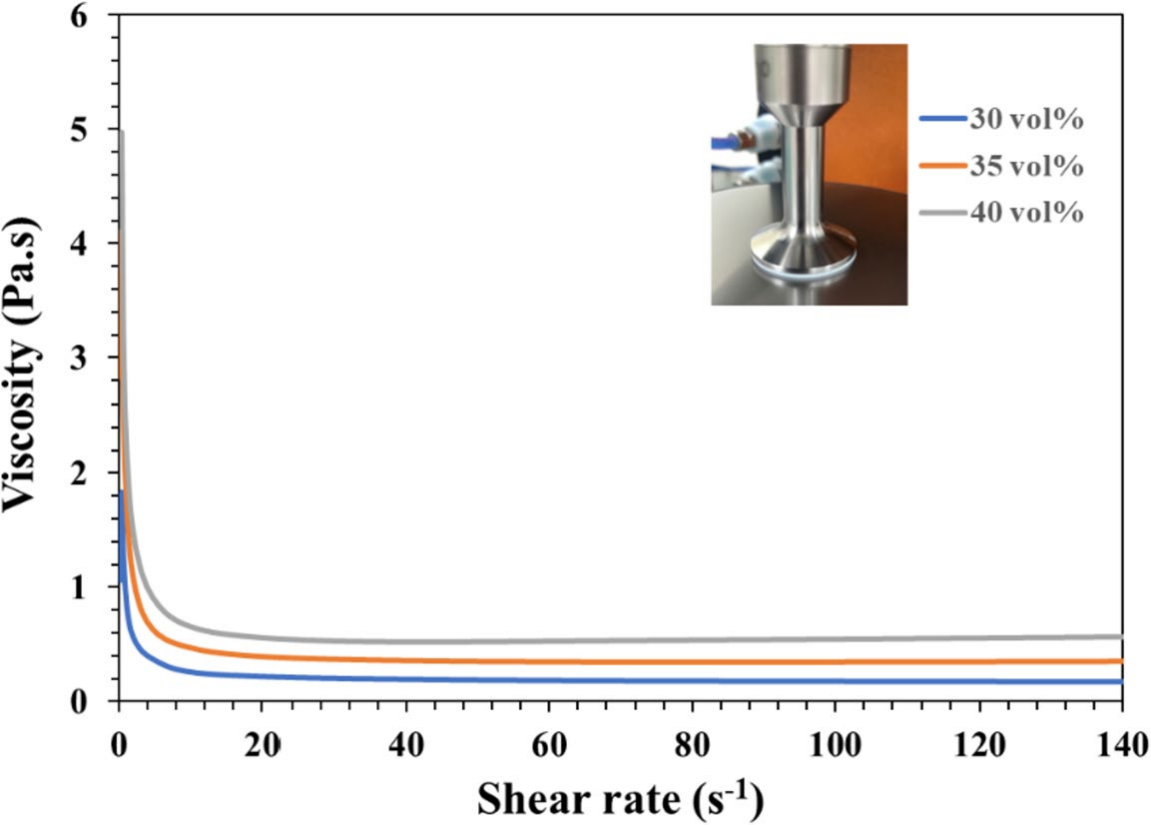
The viscosity curves of the NTR bioceramic slurry with increasing shear rate, where loaded three kinds of solid phase volume percent (30 vol%, 35 vol%, and 40 vol%)

How to prepare the low-viscosity photo-cured ceramic slurry is still a limitation or key factor of the current DLP 3D printing technology [27]. Literature showed that suitable for photo-cured 3D printing ceramic slurry generally shall have a viscosity of less than 3–5 Pa·s at a shear rate of 30 s^−1^ [27, 39]. To select an NTR ceramic slurry suitable for photo-cured printing of DLP in this study, Fig. 4 shows the results of the viscosity curves of the NTR bioceramic slurry with increasing shear rate, where three kinds of solid phase volume percent (30 vol%, 35 vol%, and 40 vol%) will be evaluated. Their results showed that the viscosity of NTR bioceramic slurry decreased with the increase in shear rate. In addition, these viscosities also only minor increase when the solids content increases from 30 vol% to 40 vol %. Among them, the viscosity was less than 3–5 Pa·s when the shear rate was greater than 5 s^−1^; it can be shown that its viscosity may meet the requirements of DLP 3D printing. Whatever, it is necessary to maintain a lower viscosity level at the beginning of action for the top-down DLP 3D printing system in our experience. In other words, if the viscosity of the NTR ceramic slurry increases by more than 3–5 Pa·s at the beginning, the successful printing under the DLP process system will easily fail. That says it will be difficult to maintain successful printing out 3D structures at the solid content level of 40 vol%. Therefore, we chose a solid content of 35 vol% as the stability parameter for the NTR bioceramic slurry to fabricate the concave-top disc sintered 3D-printed bioceramic of osteochondral graft and mechanical test sample.

### Determination of the drying condition and sintering temperature curve

After the as-received 3D-printed bioceramic green-body is obtained, the residual water in the sample must be removed by drying [40, 41]. Deformation and cracks of the sample can occur quite easily during the drying process with the traditional natural drying method [32, 40]. In particular, layer delamination and cracking can frequently occur due to the internal stress generated when materials such as solvents, residues, and polymers emerge from the objects during the drying and debinding processes [41]. Hence, it is critical to control the drying process to protect the sample from deformation and cracking. It is worth mentioning that our as-received 3D-printed bioceramic green-body can make the dehydration uniform and accelerate the drying in a 60°C oven for 1 h in this study, which is based on negative temperature responsive ceramic slurry characteristics and silicon oil enveloped show video of Supplemental information [16, 17, 20, 42].

**Fig. 5:**
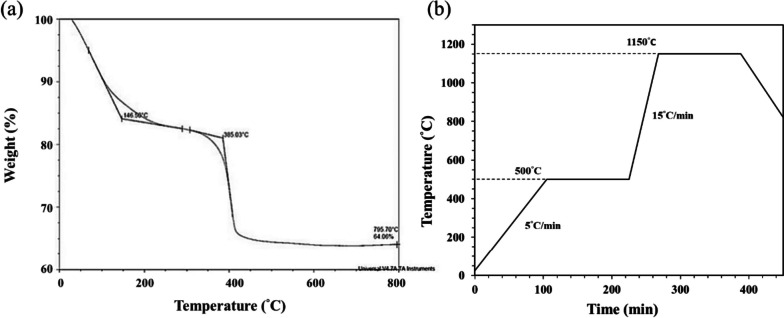
The thermogravimetric (TGA) analysis of the 3D-printed bioceramic green-body (**a**). The debinding and sintering temperature profile of 3D-printed bioceramic green-body in an atmosphere (**b**)

The last challenge for sintered 3D-printed ceramic is whether the sintering conditions can obtain a highly densified and crack-free ceramic sintered body, and studying the thermal behavior of the 3D green-body of the bioceramic specimen is one of the key steps [43–46]. Therefore, the TGA test was performed to obtain the thermal debinding/degradation temperature, and the TGA curve result of the 3D-printed bioceramic green-body is shown in Fig. 5a. The first weight loss stage should be attributed to ethanol, water, minor monomers of NIPPAm, and lower molecule weight of PEGDA (< 500 Da), etc., which evaporate from 25.0°C to 146.5°C. Then, the percent weight loss in the first stage is about 15%. The second weight loss stage still shows a vaporization reaction between 146.5°C and 385.0°C, which should be comprised of silicone oil, trimethylolpropane triacrylate (TMPTA), and average molecular weight of PEGDA (~ 700Da), etc., the weight loss percentage of the second stage is about 5%. The third weight loss stage was the stronger endothermic decomposition reaction of between 385.0℃ and 500.0℃ and should be offered by the main weight of photo-crosslinking resin polymer and minor photo-initiator of I819.

However, according to the TGA curve results, the sintering process is designed in a high temperature oven in the atmosphere, as shown in Fig. 5b. Which is mainly divided into the following two steps: the first step is to debinding and prevent the sintered specimen from cracking, and the heating rate is set from room temperature to 500°C at the rate of 5°C/min and kept at 500°C for 2 h. Subsequently, the second step is to sinter densification and enhance the mechanical properties of the sintered specimen. Therefore, the heating rate was set from 500°C to 1150°C at the rate of 15°C/min and kept at 1150°C for 2 h, and then the oven was naturally cooled to room temperature.

### Physical and chemical property analysis of sintered 3D-printed bioceramic

#### Phase structure analysis

**Fig. 6:**
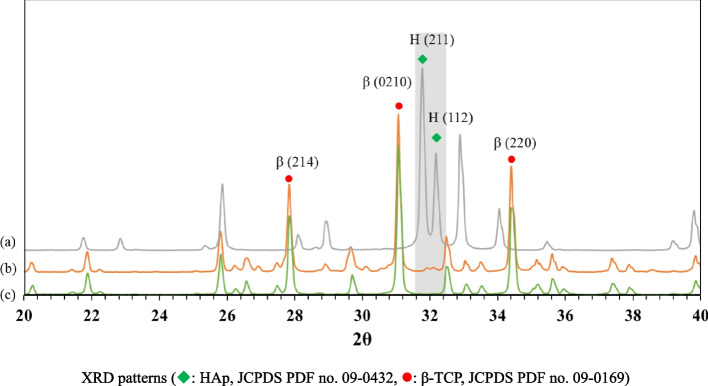
XRD patterns of as-received HAp powder (**a**), as-received β-TCP powder (**b**), and (**c**) the sintered 3D-printed bioceramic scaffolds sintered at 1150 °C for 2h

Figure 6 shows the XRD patterns of β-TCP powder, HAp powder, and the sintered 3D-printed bioceramic scaffold after sintering at 1150 ℃. The XRD peaks of all the diffraction patterns agree well with those of standard HAp and β-TCP in the powder diffraction file (standard XRD pattern of Card no. 9–432 and 9–169). However, each diffraction peak of the sintered 3D-printed bioceramic scaffold after sintering at 1150 ℃ was almost identical to β-TCP powder, such as the prominent peaks for (2, 1, 4), (0, 2, 10), and (2, 2, 0) planes of β-TCP were observed in line with the purpose of the phase structure to be achieved. The high-temperature treatment will lead to the HAp phase decomposing to β-TCP, resulting in a highly crystalline [22, 38, 47]. And the rationale for the 7/3 ratio of the β-TCP/HAp strategy wants to use an in-situ phase transition mechanism to increase its internal compressive stress. The reason is that the volume will have a small volume expansion based on the higher density of a small portion of HAp (3.16 g/cm^3^) crystal transit to the lower density β-TCP (3.14 g/cm^3^) [48].

#### The shrinkage, density and porosity analysis

Owing to the drying process should be to prevent deformation and cracks of the as-received 3D-printed bioceramic green-body. Therefore, the silicon oil film was used to seal the surface of the as-received 3D-printed bioceramic green-body can promote the uniform drainage of water to shrink these ceramic powders effectively under the 60 °C oven for 1 h in this study. This phenomenon is like vacuum packaging ceramic green bodies under envelop film in cold isostatic pressure (CIP) [22]. It should eventually make the sintered density of the sintered 3D-printed bioceramic scaffolds denser and increase the mechanical properties. However, we found that the shrinkage rate of the as-received 3D-printed bioceramic green-body from the drying and sintering process differs in the XY plane's diameter and vertical direction's height. The shrinkage analysis results in Table 1 showed that the shrinkage rate of the drying process from the diameter of XY plane and height of vertical direction is 10.2% and 5.6%, respectively. Then, the shrinkage rate of the sintering process from the diameter of the XY plane and the height of the vertical direction is 7.6% and 10.4%, respectively. Then, the XY plane diameter and vertical height shrinkage ratio between drying and sintering are very close, 17.8% and 16.0%, respectively. This means that the overall compactness of the vertical direction after sintering will also be a little bit lower than the diameter of the XY plane. Whatever, it still can be expected to avoid the serious formation of internal stresses and cracks resulting from uneven dehydration and the main photo-crosslinking polymer burn-off gases because each shrinkage rate step is slow and smaller than 12%. But, they still need to be evaluated by follow-up microscopic observation.

**Table 1:** The results of average shrinkage analysis, average bulk density, average apparent density, and total porosity of the concave-top disc sintered 3D-printed bioceramic scaffolds

Shrinkage ratio (%)				Density (g/cm^3^)		Total Porosity (%)
Drying		Sintering				
Diameter	Height	Diameter	Height	Bulk density	Apparent density	56.5 ± 0.6% (from bulk density)
10.2 ± 0.3%	5.6 ± 0.4%	7.6 ± 0.2%	10.4 ± 0.5%	1.37 ± 0.16	2.91 ± 0.12	

The apparent density was referenced to the Archimedes method of previous research [22, 49]; as previously mentioned, Eq. 1 was utilized, and the illustration in sFig. 1 of supplemental information, the average resulting in about 2.91 g/cm^3^ (compared with theoretical density up to 94.8%) as shown in Table 1. This suggests the higher degree of sintering densification achieved by the NTR-based bioceramic slurry at a relatively low ceramic powder volume of about 35 vol%. The bulk density results were also determined by the previously mentioned Eq. 2 and the illustration in sFig. 1 of supplemental information, the average resulting in about 1.37 g/cm^3^. In addition, the average bulk porosity of the concave-top disc sintered 3D-printed bioceramic scaffolds was about 56.47%. Because the bulk volume calculation of the concave-top disc sintered 3D-printed bioceramic scaffolds comprising the continuous channel volume was determined by Eq. 3, the illustration in sFig. 1 of supplemental information.

#### Microstructural observation and elemental semi-quantitative evaluation

**Fig. 7:**
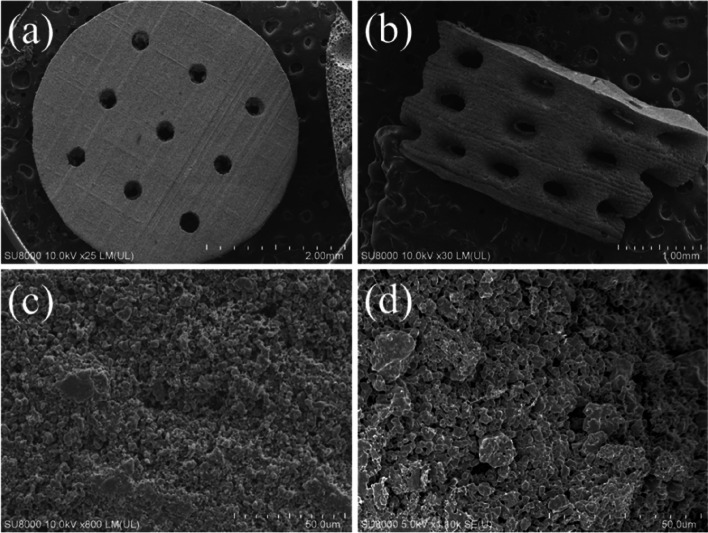
SEM morphologies of the concave-top disc sintered 3D-printed bioceramic scaffolds were evaluated, the bottom view on the x–y plane (**a**, **c**), lateral view on the side of the vertical direction (**b**, **d**)

The surface and cross-sectional microstructures of the concave-top disc sintered 3D-printed bioceramic were evaluated by SEM, as shown in Fig. 7. Morphological investigations of Fig. 7a and b showed that the 3D sintered β-TCP bioceramic was compactness on the XY plane than the lateral side of the vertical direction. Because there are microcracks between layers on the lateral side of the vertical direction, this result is consistent with the shrinkage result. It is speculated that if the interlayer polymerization time is adjusted a little longer, the monomer resin between the upper and lower layers will have a higher degree of crosslinking, which would result in better densification in the lateral side of the vertical direction [44]. In addition, Lin et al*.* also reported that increasing the photoinitiator concentration decreases print resolution due to excess radical production and diffusion [50]. In other words, the photoinitiator concentration decreases, and the penetration depth of the photons increases, where the free-radical initiation is not thus localized closer to the surface. Therefore, the depth of the light source of each layer can be increased, which could result in better densification in the lateral side of the vertical direction.

Furthermore, based on the results reported by Murphy et al. in their study, a pore size of approximately 325 μm is considered the most suitable [51]. Final interconnected pore channel consists of circular pores with a diameter of 250–400 μm on the XY plane. The lateral view showed the elliptical pores have long and short axes of 400–500 µm and 200–300 µm, respectively. And the macropore size is in the range of 100 to 800 µm, which is optimal to allow diffusion of nutrients/removal of waste byproducts, bone cell colonization, blood vessel infiltration, and new bone regeneration [52]. Regardless, the micropores smaller than 10 µm still were observed from the interparticle in the higher magnification images, as shown in Fig. 7c and d. Each grain size was distributed in the range of about 2–5 µm, and most of the grain shapes were irregular. The microstructure of sintered bioceramic surface was also shown to be closed on the XY plane than the lateral side of the vertical direction because obvious microcracks can be observed on the lateral side of the vertical direction.

In addition, the SEM–EDS has proven to be a rapid investigation tool to detect which elements are present in the processed sintered 3D-printed bioceramic scaffolds. As can be seen in Table 2, the main elements of the silicone oil-sealed sintered 3D-printed bioceramic scaffolds were C, O, Si, P, and Ca. The Ca/P molar ratio was close to 1.41. In contrast, the mole percentage of elemental silica was about 2.29 atomic%. Furthermore, the increase of 2.29 atomic% silicon element from silicone oil-sealed 3D-printed bioceramic scaffolds was expected to be consistent with the current results in the kinds of literature, suggesting that silicate ions could be enhanced vascularization and osteogenic differentiation *in vitro* and *in vivo* [53], then promote repair of two different differentiated tissues in vivo (cartilage and bone) [54].

**Table 2:** The results of elemental analysis of the concave-top disc sintered 3D-printed bioceramic scaffold by SEM–EDS

Element	C	O	Si	P	Ca	Ca/P ratio
Weight %	5.29	39.28	2.83	18.59	34.01	–-
Atomic %	8.80	55.91	2.29	13.67	19.33	1.41

#### Compression strength evaluation

To analyze the compression strength of the sintered 3D-printed bioceramic scaffolds, typical compression data profiles are shown in sFig. 2 of the supplemental information using the initial external dimensions (ϕ 4.8 × height 12.0 mm) of each sample. The average compressive strength can reach 11.38 $\pm$ 1.72 MPa. Therefore, the silicon oil film was used to seal the surface of the as-received 3D-printed bioceramic green-body to promote the uniform drainage of water to shrink these ceramic powders effectively under the 60 °C oven for 1 h. In addition, the overall shrinkage of the XY plane diameter and vertical height from the as-received 3D-printed bioceramic green-body to drying and sintering is about 17.8% and 16.0%, respectively. The newly developed denser sintered 3D printed bioceramic (β-TCP) scaffold process can be made using DLP technology and the novel photocurable negative thermo-responsive (NTR) bioceramic slurry. Furthermore, sealing the surface of the as-received 3D-printed bioceramic green-body with the silicon oil cover can avoid the serious formation of internal stresses and cracks resulting from uneven dehydration. Finally, better mechanical properties can be obtained after a proper sintering profile.

#### Cytotoxicity of 3D-printed bioceramic

**Fig. 8:**
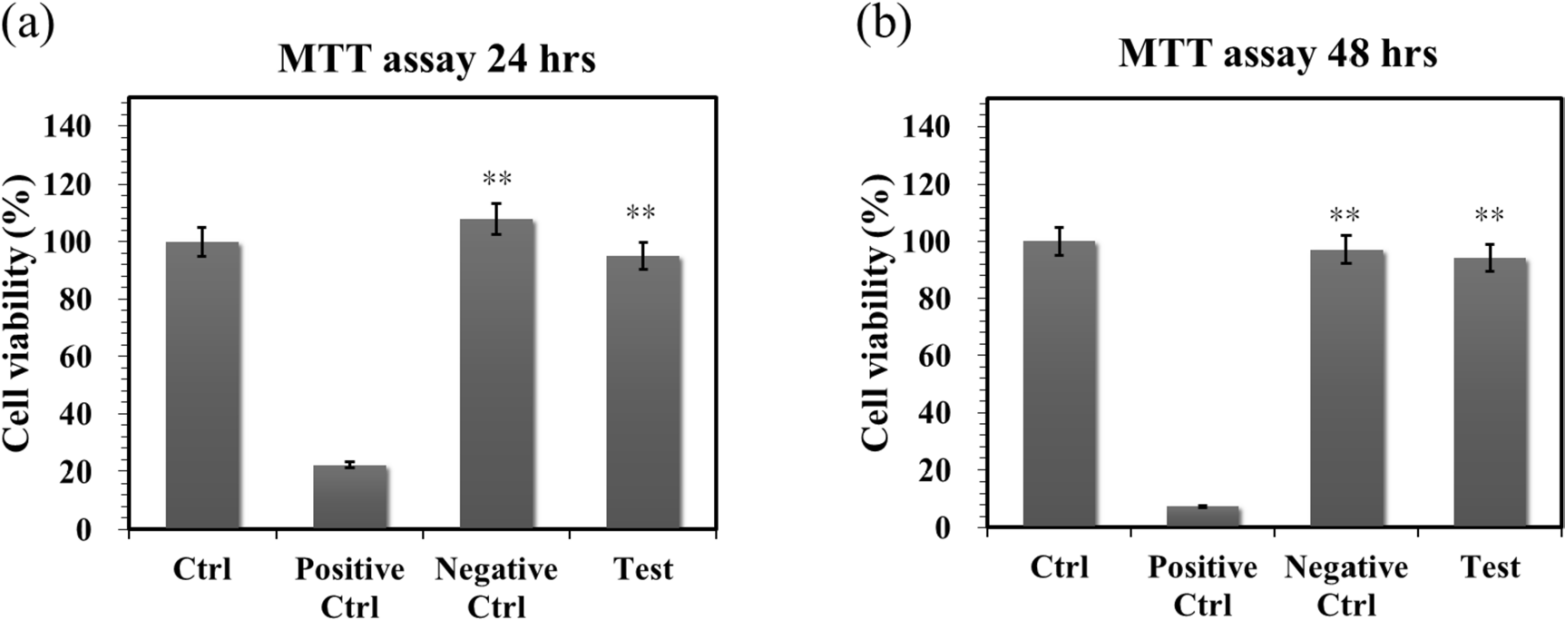
The cell viability of NIH-3T3 cells was evaluated by MTT assay on the sintered 3D-printed bioceramic scaffolds at 24 and 48 h. The SDS is used as a positive control, and a group consisting exclusively of cells and a group with only Al_2_O_3_ powders were used as blank controls and negative controls. The error bar represents ± SD (*n* = 6); *p < 0.05 and **p < 0.01 compared with the positive control group

Biocompatibility is a major requirement for implanted bilayer osteochondral graft substitutes. We have approved that the photo-cured hybrid biohydrogel system (HG + 0.5AFnSi) is non-cytotoxic and can provide a suitable environment for the chondrogenesis of hADSCs in the previous report [18]. However, the subchondral scaffolds of the sintered 3D-printed bioceramic (β-TCP) scaffolds still need to be further evaluated for cytotoxicity. Therefore, we assessed cell viability by the MTT assay following ISO 10993–5. The results in Fig. 8a and b show that these sintered 3D-printed bioceramic scaffolds exhibit well cell viability for NIH-3T3 cells after 24 h and 48 h of cell culture. For example, the blank controls are averaged and set to 100% cell viability by OD450-630 absorbance readings at 24 and 48 h. The following proportion of the negative control, positive control, and test extracts at 24 h are 22.18%, 108.00%, and 95.01%, respectively. And then, the results of cell viability at 48 h also indicated the positive control, negative control, and test extracts to be 7.38%, 97.12%, and 94.27%, respectively. Anyway, the negative control and test groups showed no significant changes in cell morphology compared to the blank group, while the positive control group showed severe growth inhibitory cells. Above cytotoxicity evaluation results show that the sintered 3D β-TCP bioceramic scaffolds with minor silicon elements (< 3 Atomic %) also have good cell biocompatibility.

### The in vivo regeneration of osteochondral defects in rabbit

#### Soft X-ray radiograph analysis

**Fig. 9:**
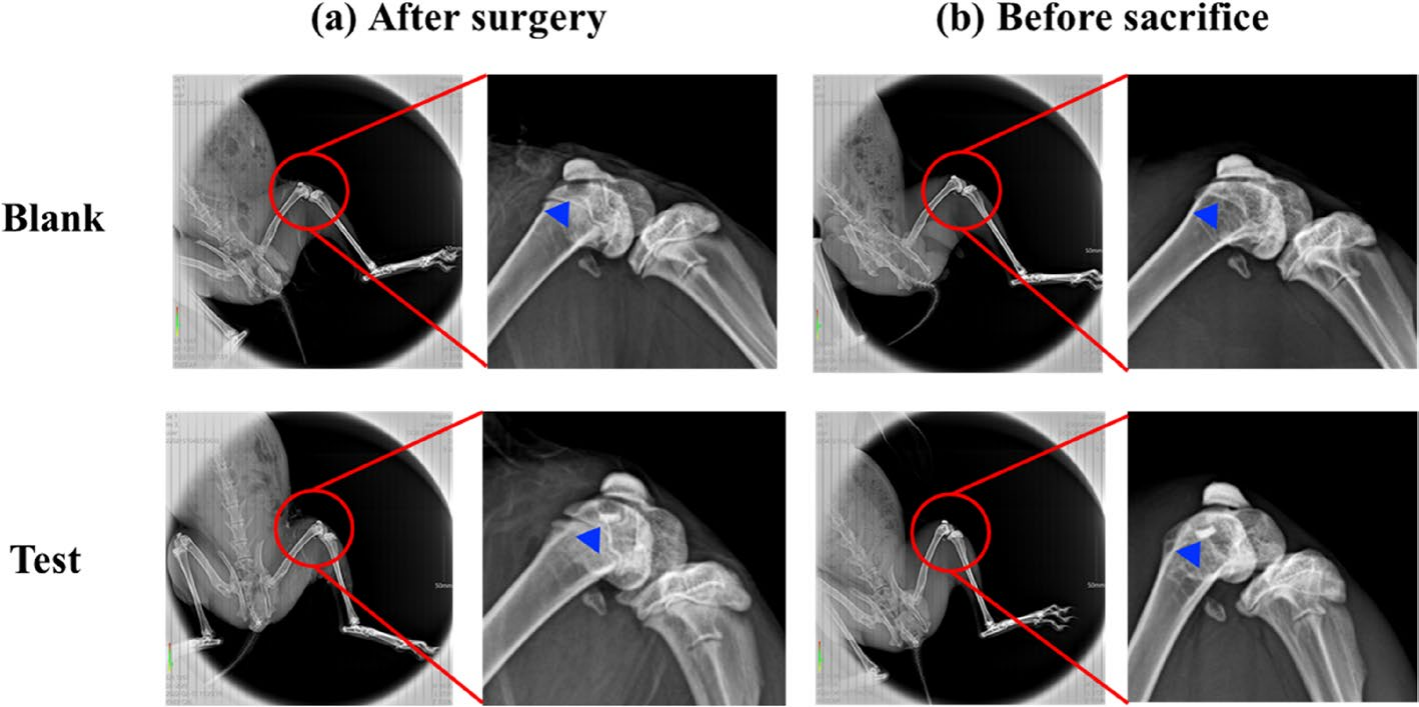
Soft X-ray photographs of osteochondral defect of femoral trochlear in rabbit cartilage blank and experimental groups. Osteochondral defects immediately after the surgery (**a**) Osteochondral repair defect before sacrifice (**b**)

As calcium phosphate is naturally radiopaque, especially all the dense sintered 3D-printed bioceramic scaffolds are more thus radiopaque, being visible on X-ray images as soon as they are until they are resolved and substituted by newly formed bone. The radiographs taken immediately after surgery clearly revealed osteochondral defects in all the animals. However, the sintered 3D-printed bioceramic scaffolds are still visible in the defects of the test groups, shown in sFig. 4 (b) (blue delta) at 8 weeks of supplemental information and Fig. 9 (b) (blue delta) at 12 weeks. Before sacrifice for 8 weeks and 12 weeks, it was evident that a blurred outline of osteochondral defect surrounded the defect site in the blank group. However, the chondral defect area in test groups also appeared blurry between after surgery and before sacrifice, as shown in sFig. 4 (a), (b) and Fig. 9 (a), (b) (blue delta). Preliminary radiographic examinations showed satisfactory results with low degradation rates for all samples and maybe a positive effect on the healing of the osteochondral defect. These findings suggest that the sintered 3D-printed bioceramic scaffolds with a stiff subchondral bony compartment would provide stable mechanical support for cartilage regeneration and enhance subchondral bone regeneration. The success of cartilage repair is closely related to the integrity of the subchondral bone tissue, and the primary purpose of the bioceramic scaffold is to provide support for cartilage growth and the environment for bone growth [55]. Therefore, as long as the degradation rate of the bone layer of the osteochondral scaffold is slower than that of the cartilage scaffold, it is considered acceptable in clinical practice.

#### Gross examination and histological examination

In addition to examining whether hADSCs laden with the novel photo-cured hybrid biohydrogel system (HG + 0.5AFnSi) of the bilayer osteochondral graft can provide a suitable environment for the regeneration ability of osteochondral defect in the rabbit knee joint. It is worth observing whether 3D-printed bioceramic scaffolds of the bilayer osteochondral graft have the following advantages in osteochondral defect sites: (1) Whether the sintered 3D β-TCP scaffolds are expected to provide long-term stable support for the subchondral substrate can better restore the integrity of the osteochondral tissue. (2) Because the dense middle layer of the sintered 3D β-TCP scaffold separates ADSCs and BMSCs within the bilayer osteochondral graft. Does it affect the ability of the chondral layer to regenerate as a result? (3) Whether the sintered 3D β-TCP scaffold can promote the tissue regeneration of subchondral trabecular bone and whether the sintered 3D β-TCP scaffold will also gradually degrade and be replaced by autologous bone.

**Fig. 10:**
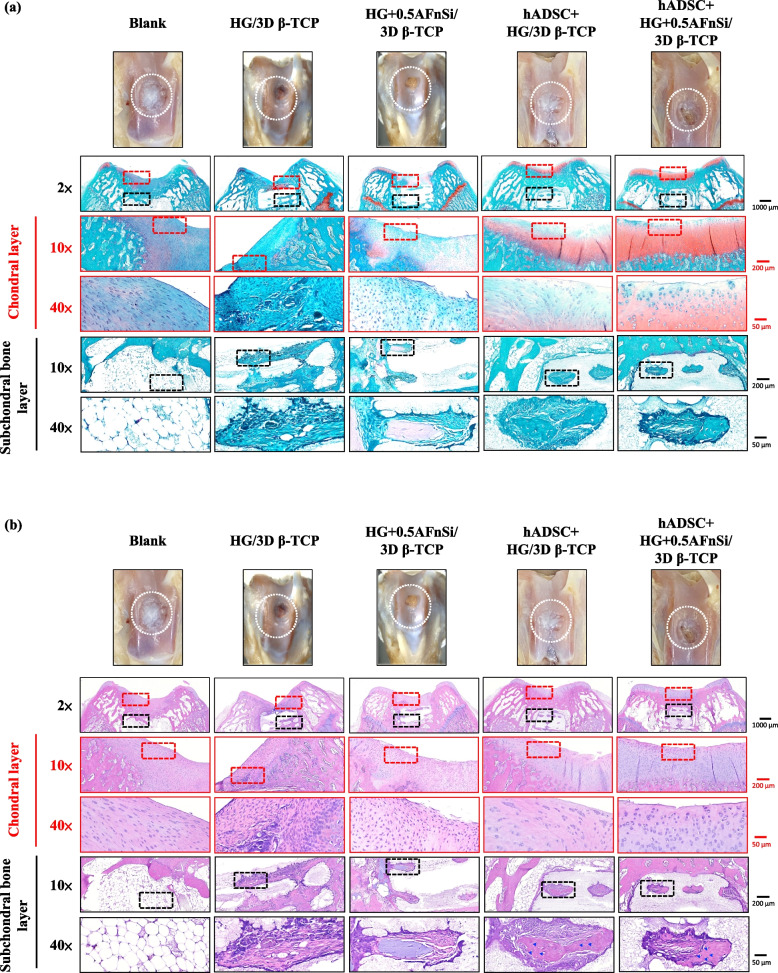
After 8 weeks of surgery, the gross examinations were inspected by optical photos, and the biological and chemical composition of bilayer osteochondral grafts were assessed. The glycosaminoglycan (GAG) distribution analysis using Safranin-O staining (**a**). The HE stating is hematoxylin staining the nuclei in blue-purple, while eosin staining stains the cytoplasm and extracellular matrix pink (**b**). Red dashed line box: the chondral layer defect area; Black dashed line box: the subchondral layer defect area; Blue delta showed osteocyte

In the first-phase in vivo study, the visual inspection results of the appearance of the repaired osteochondral defect 8 weeks after surgery are shown in Fig. 10. Some neo-tissue formation of spontaneous regeneration was observed in the blank group, HG without hADSCs of the bilayer osteochondral graft (HG/3D β-TCP) group, and HG + 0.5% AFnSi without hADSCs of the bilayer osteochondral graft (HG + 0.5AFnSi/3D β-TCP) group. At the same time, some white newly grown tissues appeared in the HG with hADSCs of the bilayer osteochondral graft (hADSC + HG/3D β-TCP) group and HG + 0.5% AFnSi with hADSCs of the bilayer osteochondral graft (hADSC + HG + 0.5AFnSi/3D β-TCP) group. However, the defect area was significantly reduced while well-integrated glossy white tissues were detected. The osteochondral defect in all groups still had not healed well at the 8 weeks, and the osteochondral defect site still had a little defect area that failed to cover the defect surface.

Subsequently, these histological specimens’ groups comprising of the blank, HG/3D β-TCP, HG + 0.5AFnSi/3D β-TCP, hADSC + HG/3D β-TCP, and hADSC + HG + 0.5AFnSi/3D β-TCP were obtained at 8 weeks after surgery and subjected to Safranin-O staining and HE staining in Fig. 10 a and b. The Safranin-O staining could appear red–orange and stains proteoglycans (PGs) present in the extracellular cellular matrix. The staining intensity reflects the number of PGs present, with darker staining indicating higher concentrations of PGs [56–58]. I In contrast, to responsible for the new bone formation at the subchondral layer, and Safranin-O staining is not typically used since the bone tissue does not contain PGs in the same way as cartilage. The first-phase group's results of the integrity of the morphology and thickness of the cartilage-like layer were assessed using the Safranin-O staining and shown in Fig. 10a. Among them, the PGs concentrations of both groups with hADSCs are better than those without hADSCs, such as the hADSC + HG/3D β-TCP group and hADSC + HG + 0.5AFnSi/3D β-TCP group. And although the blank group and HG + 0.5AFnSi/3D β-TCP group without hADSCs seem to have more neo-tissue formation, the response from PGs is still less. Even though the defect area was significantly reduced while well-integrated glossy white tissues were detected, the formation of PGs in the extracellular matrix of the cartilage layer was still not seen in the two groups of HG-based osteochondral grafts without hADSCs and the blank group.

In addition to characterizing the ability of cartilage regeneration in these areas according to the biochemical components of the ECM as described above, further observation of the cell morphology and number between the cartilage layer and the subchondral layer can also be used as a screening condition for osteochondral regeneration [59, 60]. It is known from some of the literature that the morphology and number of chondrocytes in different regions of the knee cartilage layer are as follows [59, 61, 62]: the superficial zone is the outermost and thinnest of all layers of the joint cartilage. It is composed of a relatively high number of oval-shaped chondrocytes parallel to the surface of the joint [59, 61, 62]. The chondrocytes of the middle (or transitional) zone are spherical and have low density. And then, Chondrocytes in the deep zone are larger and are usually available in a columnar orientation and perpendicular to the surface [59, 61, 62]. The calcified cartilage zone plays an integral role in the fixation of cartilage to the bone by anchoring collagen fibrils from the deep zone to the subchondral bone. In this zone, the cell population is scarce, and chondrocytes are hypertrophic, which makes metabolic activity very low [59, 61, 62].

Therefore, the morphological and number analysis of chondrocytes in different areas of the chondral layer and subchondral trabecular bone at 8 weeks after surgery were shown in HE staining of Fig. 10b. Owing to hematoxylin stains nuclei blue-purple, while eosin stains the cytoplasm and extracellular matrix pink [63, 64]. Therefore, they appear blue-purple could be the presence of hADSCs and chondrocytes in the extracellular matrix for the chondral layer and the presence of BMSCs/osteoclasts/osteoblasts/bone cells in the extracellular matrix for the subchondral trabecular bone were shown in Fig. 10b. Besides, it also can appear pink due to the cytoplasm and the surrounding extracellular matrix for the chondral and subchondral layers. To check the morphology of chondrocytes from superficial to deep areas of the chondral layer of Fig. 10b. Only hADSC + HG/3D β-TCP group and hADSC + HG + 0.5AFnSi/3D β-TCP group had similar natural cartilage morphology when the chondral layer was regenerated due to the presence of hADSCs. In particular, the group of hADSC + HG + 0.5AFnSi/3D β-TCP showed the highest number of cells. In other words, a relatively high number of oval-shaped chondrocytes appeared parallel to the joint's surface; the chondrocytes are spherical and have a low density in the middle zone. And then, chondrocytes are larger and are usually available in a columnar orientation and perpendicular to the surface in the deep zone. The morphology of these hADSCs differentiated into chondrocytes is consistent with the analysis results of PGs in Fig. 10a. In addition, the blank group and the other two groups of HG-based without hADSCs of the bilayer osteochondral graft, whose histological cell morphology showed non-chondrocytes in the chondral layer and may be derived from the synovial fluid or BMSCs in the bone marrow. Among them, the obvious defect appearance on the surface of HG/3D β-TCP may be the crosslinking not enough of its HG hydrogel.

Next, we are going to examine the regeneration of the cancellous bone area under the chondral layer. The results in Fig. 10b show that the blank group has no obvious collapse phenomenon. However, histological observations showed that the self-regeneration ability of the chondral layer was poor, the subchondral trabecular bone area was empty, and the repaired subchondral tissue was disordered bone tissue. The other groups of the bilayer osteochondral grafts further observe cell morphology within the subchondral trabecular bone area in Fig. 10b. It can be seen that the interconnected pore channels of a 3D-printed bioceramic scaffold can allow BMSCs and bone cells to conduct new bone formation after implant surgery. In addition, the structure of the 3D sintered β-TCP scaffolds remained stable, and the subchondral trabecular bone regions still showed no obvious degradation before 8 weeks. Although β-TCP belongs to the phase structure of in vivo biodegradation, this is attributed to the high densification of the 3D sintered bioceramic structure in this study, which also has stronger crystallinity, so it can resist rapid degradation in vivo.

**Fig. 11:**
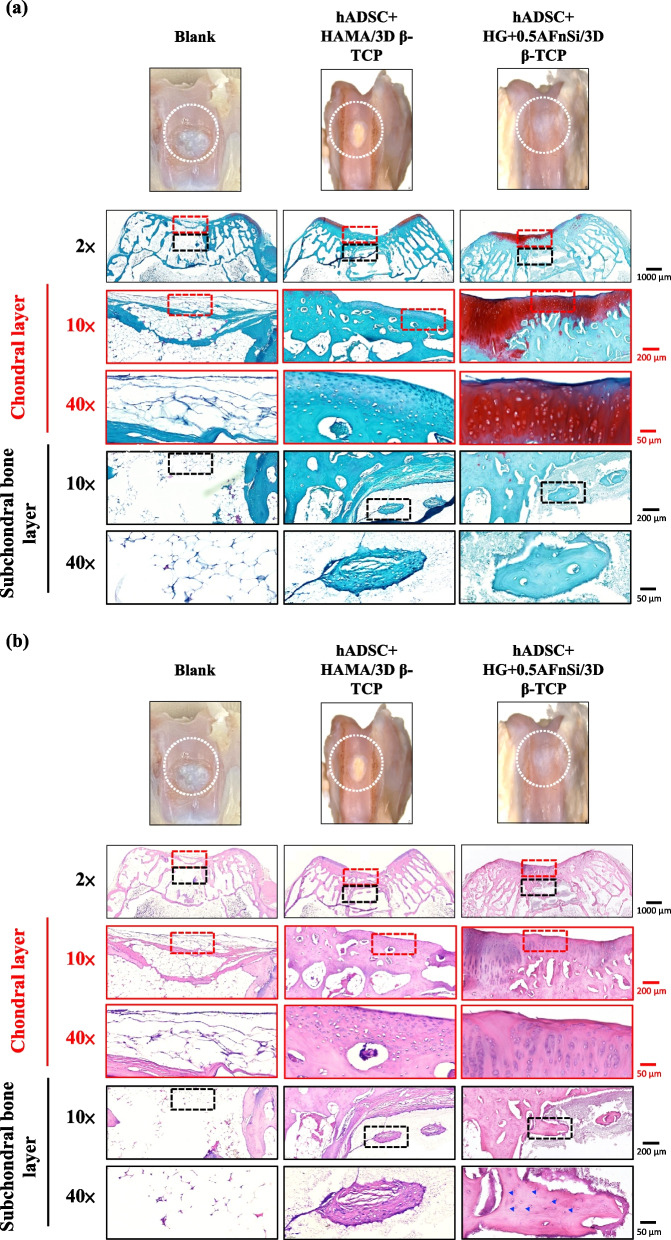
After 12 weeks of surgery, the gross examinations were inspected by optical photos, and the biological and chemical composition of bilayer osteochondral grafts were assessed. The glycosaminoglycan (GAG) distribution analysis using Safranin-O staining (**a**). The HE stating is hematoxylin staining the nuclei in blue-purple, while eosin staining stains the cytoplasm and extracellular matrix pink (**b**). Red dashed line box: the chondral layer defect area; Black dashed line box: the subchondral layer defect area; Blue delta showed osteocyte

Because the groups of hADSC + HG + 0.5AFnSi/3D β-TCP have better repair and regeneration performance of osteochondral defects for 8 weeks. In the second phase in vivo study at 12 weeks of animal experiment, we maintained the blank and hADSC + HG + 0.5AFnSi/3D β-TCP group experimental groups. In addition, a group of positive control of HAMA with hADSCs of the bilayer osteochondral graft (hADSC + HAMA/3D β-TCP) was added for animal experiment comparison of osteochondral defect regeneration. The visual inspection results of the appearance of the repaired osteochondral defect 12 weeks after surgery are shown in Fig. 11. The more neo-tissue formation of spontaneous regeneration was still observed in the blank group. Only, it appears more transparent tissue than other groups and normal cartilage surface. However, the positive group of HAMA/3D β-TCP also showed good coverage repair. Still, the defect area's growing tissue was whiter and more opaque than the surrounding normal cartilage area. However, results with better osteochondral defect regeneration showed that in the group of hADSC + HG + 0.5AFnSi/3D β-TCP since newly grown tissues of glossy white can appear. Therefore, the osteochondral defect in all groups seems healed and covered the defect area at 12 weeks; just the osteochondral defect site had a little bit of color and difference in transparency.

And then, the Safranin-O staining and HE staining for histological samples evaluation also continued at 12 weeks after surgery, and the results are shown in Fig. 11a and b. The Safranin-O staining results of Fig. 11a showed the morphology and thickness of the cartilage-like layer. The proteoglycans (PGs) concentrations of the group of hADSC + HG + 0.5AFnSi/3D β-TCP is still better than blank groups and hADSC + HAMA/3D β-TCP group. That is, the red–orange stains proteoglycans with darker staining indicating higher concentrations, which staining intensity reflecting the amount of proteoglycans present. In addition, although the blank group and the hADSC + HAMA/3D β-TCP group seem also to have more neo-tissue formation, the response from GAGs is still low amount or none.

Subsequently, the morphological and number analysis of chondrocytes in different areas of the chondral layer and subchondral trabecular bone 12 weeks after surgery was shown in the HE staining of Fig. 11b. To check the morphology of chondrocytes from superficial to deep areas of the chondral layer. The group of hADSC + HG + 0.5AFnSi/3D β-TCP was still the one with the most similar natural cartilage morphology when the chondral layer regenerated for 12 weeks due to the presence of hADSC + HG + 0.5AFnSi of the bilayer osteochondral scaffold. The group of hADSC + HG + 0.5AFnSi/3D β-TCP showed that the number of cells is the highest, and the chondrocyte morphology of each partition in the cartilage layer was closest to the natural cartilage layer. In other words, a relatively high number of oval-shaped chondrocytes appeared parallel to the joint's surface. The chondrocytes are spherical and have a low density in the middle zone. And then, chondrocytes are larger and are usually available in a columnar orientation and perpendicular to the surface in the deep zone.

Then continue in Fig. 11b examines the regeneration of the trabecular bone area under the chondral layer at 12 weeks after surgery. It also can be seen that the blank group without 3D-printed bioceramic scaffolds has no obvious collapse phenomenon. However, histological observations of the blank group showed that the self-regeneration ability of the chondral layer was still poor, and the cancellous bone area was empty too. The other two groups of the bilayer osteochondral grafts (hADSC + HG + 0.5AFnSi/3D β-TCP and hADSC + HAMA/3D β-TCP) further to observe cell morphology within the subchondral trabecular bone area in Fig. 11b, it can be seen that the interpenetrating pore channels of 3D-printed bioceramic scaffold can have more new bone formation after implant surgery at 12 weeks. Regardless, the structure of the sintered 3D-printed β-TCP scaffold was still mostly preserved. However, the subchondral trabecular region showed more degeneration at 12 weeks than at 8 weeks. It did not demonstrate host-graft rejection from hADSCs laden with biohydrogel bilayer of osteochondral graft in the rabbit xenogeneic transplantation model from multi-nucleated cells (macrophages).

**Fig. 12:**
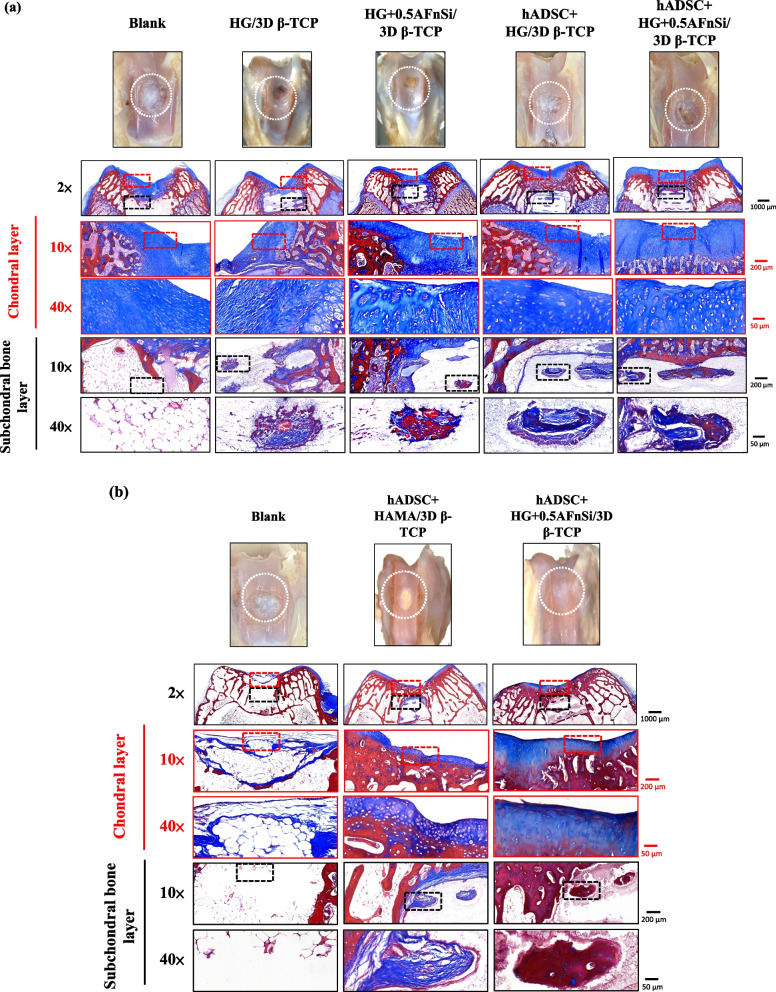
Optical photos inspected the gross examinations, and the collagen composition of bilayer osteochondral grafts was assessed using Masson’s trichrome staining. At 8 weeks after surgery (**a**). At 12 weeks after surgery (**b**). Red dashed line box: the chondral layer defect area; Black dashed line box: the subchondral layer defect area

In addition, we also confirmed the type II collagen staining and Masson's trichrome staining analysis for the bilayer osteochondral grafts at the 12-week, as demonstrated in sFig 3 and Fig. 12. It's worth noting that Masson's trichrome staining allowed us to define the bone and cartilage tissues effectively [65]. The collagen fibers in cartilage are typically thinner and more uniformly distributed in chondrogenic tissue, and they can appear blue [66]. Besides, the bone tissue has thicker and more irregularly distributed collagen fibers in bone tissue, and it will appear more reddish in color [67, 68]. The results of Masson’s trichrome staining at 8 weeks and 12 weeks are shown in Fig. 12. The lesion sites of the subchondral trabecular bone area were still visible histologically in all sample repair groups at 8 weeks of Fig. 12a. And the trabecular bone tissue repair of the blank group from the adjacent subchondral trabecular bone layer within the border is essentially empty. However, Fig. 12a can show that the appearance of the sintered 3D-printed bioceramic scaffold is still clearly visible in the subchondral trabecular bone area. In other words, the sintered 3D-printed bioceramic scaffolds had not been clearly degraded and transformed into the autologous cancellous bone at 8 weeks after surgery. However, the results of Masson’s trichrome staining indicated that the lesion sites of the bilayer osteochondral graft had gradually degraded and regenerated in the repair group of hADSC + HG + 0.5AFnSi/3D β-TCP at 12 weeks after surgery and shown in Fig. 12b. In particular, the border of the repair subchondral trabecular bone tissue from the adjacent layer could indicate that the 3D sintered bioceramic scaffold had an apparent transformation into autologous cancellous bone tissue. However, Masson’s trichrome staining also showed pale blue staining in all groups at 8 weeks but darker blue staining in the experimental groups at 12 weeks, indicating the presence of more collagen fiber distribution in the experimental groups. In general, the integration of the hADSC + HG + 0.5AFnSi/3D β-TCP group of the bilayer osteochondral scaffold into the defect site has good results from Fig. 12b.

Masson’s trichrome staining indicated that the bilayer osteochondral scaffold showed improved osteochondral regeneration and integration, consistent with the Safranin-O and H&E staining results. It should be noted that the enhanced bone tissue turnover by the bilayer osteochondral scaffold was observed only in the subchondral bone region, demonstrating the essential role of the sintered 3D β-TCP in promoting new bone formation. In accordance with the Safranin-O staining results, it was observed that the bilayer osteochondral graft led to the formation of a border for tissue calcification beneath the superficial zone, which helped in maintaining an avascular environment of cartilage tissue.

In comparison, the HG + 0.5AFnSi with hADSCs of the bilayer osteochondral graft showed a much better osteochondral defect repair outcome. A thicker cartilage layer with a smooth surface and uniformly aligned chondrocytes were observed. A more amount of regenerated trabecular bones at the subchondral bone layer also demonstrated better tissue integration.

One of the reasons for this phenomenon should be that we have adopted an osteochondral scaffold design in which the upper hybrid biohydrogel layer of hADSC + HG + 0.5AFnSi and the other layer of subchondral trabecular bone of the sintered 3D β-TCP are separated. Then, the dense middle layer of β-TCP can avoid each other interference between the BMSCs of the subchondral bone of the knee joint and hADSC + HG + 0.5AFnSi of the bilayer osteochondral grafts. Literature research shows that the chondrocytes differentiated from BMSCs are often fibrocartilage rather than hyaline cartilage [69, 70]. Another possible reason that needs to be explained is that the main function of the acrylate-functionalized nano-silica (AFnSi) crosslinker in the chondral layer should be to enhance the mechanical properties of its hybrid biohydrogel, leading to the ability to enhance cartilage regeneration [71]. Therefore, there is no answer to whether it is because of the effect of minor silicon on cartilage ability. In addition, this study has not also discussed whether the subchondral scaffold of the sintered 3D β-TCP scaffolds with minor silicon elements (< 3 Atomic %) can promote bone repair. Still, it will not cause the phenomenon of inhibiting bone regeneration.

## Conclusion

A well-integrated bilayer osteochondral graft was successfully fabricated in this study, which the concave-top disk sintered 3D-printed bioceramic (β-TCP) scaffold was made using digital light processing (DLP) technology and the photocurable negative thermo-responsive (NTR) bioceramic slurry; then incorporates the ADSCs laden photo-cured hybrid biohydrogel comprised of HAMA, GelMA, and 0.5% (w/v) AFnSi crosslinker on the chondral layer. The relevant essential achievements of bilayer osteochondral graft are as follows: the solid content of 35 vol% is the stability parameter of viscosity for the NTR bioceramic slurry to fabricate the concave-top disc sintered 3D-printed bioceramic of osteochondral graft and mechanical test sample. The as-received 3D-printed bioceramic green-body can make the dehydration uniform and accelerate the drying at 60°C oven for 1 h, based on NTR ceramic slurry characteristics and silicon oil enveloped. The sintering process is designed in a high-temperature range for the atmosphere; the first step is to debinding and prevent the sintered specimen from cracking, and the heating rate is set from room temperature to 500°C at the rate of 5°C/min and kept at 500°C for 2 h; the second step of the heating rate was set from 500°C to 1150°C at the rate of 15°C/min and kept at 1150°C for 2 h. However, the sintered 3D β-TCP compartment provides essential mechanical support for cartilage regeneration in the long term and slow biodegradation; the apparent density and compressive strength of the sintered 3D β-TCP can be obtained for ~ 94.8% theoretical density and 11.38 ± 1.72 MPa, respectively. Moreover, the histological examination results on the Safranin-O staining, H&E staining, and Masson’s trichrome staining of the bilayer osteochondral graft from hADSC + HG + 0.5AFnSi/3D β-TCP group can prove the promotion of in vivo repair. Healthier neocartilage with a smooth surface and uniformly aligned chondrocytes were observed in hADSCs laden with the novel photo-cured hybrid biohydrogel system 12 weeks after surgery. In addition, the more regenerated trabecular bone tissue also showed in the subchondral bone area of the osteochondral defect model. And the border of the repair subchondral trabecular bone tissue from the adjacent layer could indicate that the 3D sintered bioceramic scaffold had an apparent transformation into autologous cancellous bone tissue.

### Supplementary Information

**Additional file 1: sFig. 1.** The calculation formula and cartoon illustration of the bulk density and apparent density of the concave-top disc sintered 3D-printed bioceramic scaffolds. **sFig. 2.** A universal testing machine determined stress–strain curves of compression of sintered 3D-printed bioceramic tested specimens. **sFig. 3.** Type II collagen analyses of repaired Osteochondral tissue. **sFig. 4.** Soft X-ray photographs of osteochondral defect of femoral trochlear in other typic rabbit cartilage experimental groups. Osteochondral defects immediately after the surgery (a) Osteochondral repair defect before sacrificing at 8 weeks (b). Video. The negative temperature-responsive (NTR) bioceramic characteristics exhibit drainage shrinkage effects over a temperature range of 29°C to 60°C. Without silicon oil enveloped (left sample) and with silicon oil enveloped (right sample).

## Data Availability

The authors confirm that the data supporting the findings of this study are available within the article and its supplementary materials.
