# Supplementary material for: Bilayer osteochondral graft in rabbit xenogeneic transplantation model comprising sintered 3D-printed bioceramic and human adipose-derived stem cells laden biohydrogel
Source: J Biol Eng. 2023 Nov 27;17:74. doi: 10.1186/s13036-023-00389-x (PMC10680339; doi:10.1186/s13036-023-00389-x)
Supplement: Supplementary file 1 — Additional file 1: sFig. 1. The calculation formula and cartoon illustration of the bulk density and apparent density of the concave-top disc sintered 3D-printed bioceramic scaffolds. sFig. 2. A universal testing machine determined stress–strain curves of compression of sintered 3D-printed bioceramic tested specimens. sFig. 3. Type II collagen analyses of repaired Osteochondral tissue. sFig. 4. Soft X-ray photographs of osteochondral defect of femoral trochlear in other typic rabbit cartilage experimental groups. Osteochondral defects immediately after the surgery (a) Osteochondral repair defect before sacrificing at 8 weeks (b). Video. The negative temperature-responsive (NTR) bioceramic characteristics exhibit drainage shrinkage effects over a temperature range of 29°C to 60°C. Without silicon oil enveloped (left sample) and with silicon oil enveloped (right sample). [file 13036_2023_389_MOESM1_ESM.docx]

**Bilayer osteochondral graft in rabbit xenogeneic transplantation model comprising sintered 3D-printed bioceramic and human adipose-derived stem cells laden biohydrogel**

Chih-Yun Lee^1,2,3^, Swathi Nedunchezian^2,3,4^, Sung-Yen Lin^2,3,5,6,7^, Yu-Feng Su^8,9^, Che-Wei Wu^2,3^, Shun-Cheng Wu^2,3,10^, Chung-Hwan Chen^2,3,5,6,7,11^, Chih-Kuang Wang ^1,2,3,4,12, *^

^1^ Ph.D. program in Life Sciences, College of Life Science, Kaohsiung Medical University, Kaohsiung 80708, Taiwan

^2^ Regenerative Medicine and Cell Therapy Research Center, Kaohsiung Medical University, Kaohsiung 80708, Taiwan

^3^ Orthopaedic Research Center, College of Medicine, Kaohsiung Medical University, Kaohsiung 80708, Taiwan

^4^ Department of Medicinal and Applied Chemistry, College of Life Science, Kaohsiung Medical University, Kaohsiung 80708, Taiwan

^5^ Departments of Orthopaedics, School of Medicine, College of Medicine, Kaohsiung Medical University, Kaohsiung 80708, Taiwan

^6^ Department of Orthopaedics, Kaohsiung Medical University Hospital, Kaohsiung Medical University, Kaohsiung 80708, Taiwan

^7^ Department of Orthopaedics, Kaohsiung Municipal Ta-Tung Hospital, Kaohsiung Medical University, Kaohsiung 80145, Taiwan

^8^ Faculty of Post-Baccalaureate Medicine, College of Medicine, Kaohsiung Medical University, Kaohsiung 80756, Taiwan

^9^ Division of Neurosurgery, Department of Surgery, Kaohsiung Medical University Hospital, Kaohsiung Medical University, Kaohsiung 80708, Taiwan

^10^ Post-Baccalaureate Program in Nursing, Asia University, Taichung 41354, Taiwan

^11^Ph.D. Program in Biomedical Engineering, College of Medicine, Kaohsiung Medical University, Kaohsiung 80708, Taiwan

^12^Graduate Institute of Medicine, College of Medicine, Kaohsiung Medical University, Kaohsiung 80708, Taiwan

* Corresponding author: Chih-Kuang Wang

E-mail address: ckwang@kmu.edu.tw (C.K. Wang)

Tel.: 886-7-3121101 ext. 2677; Fax: 886-7-3125339

**
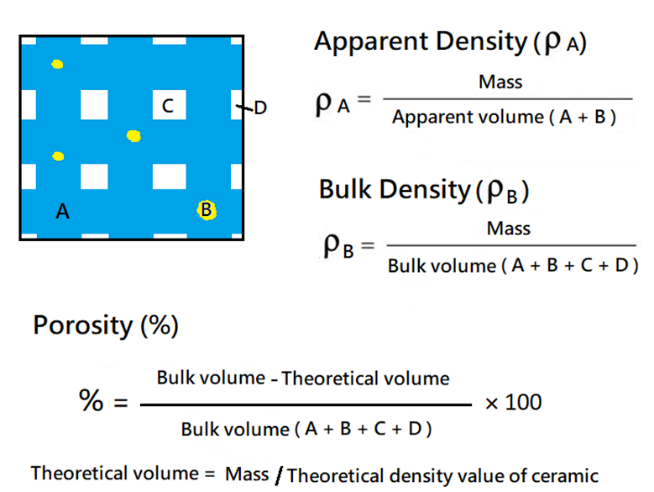
**

Note: A=solid volume, B=closed pore volume, c=middle open pore volume, D=outer open pore volume

**sFig. 1** The calculation formula and cartoon illustration of the bulk density and apparent density of the concave-top disc sintered 3D-printed bioceramic scaffolds.


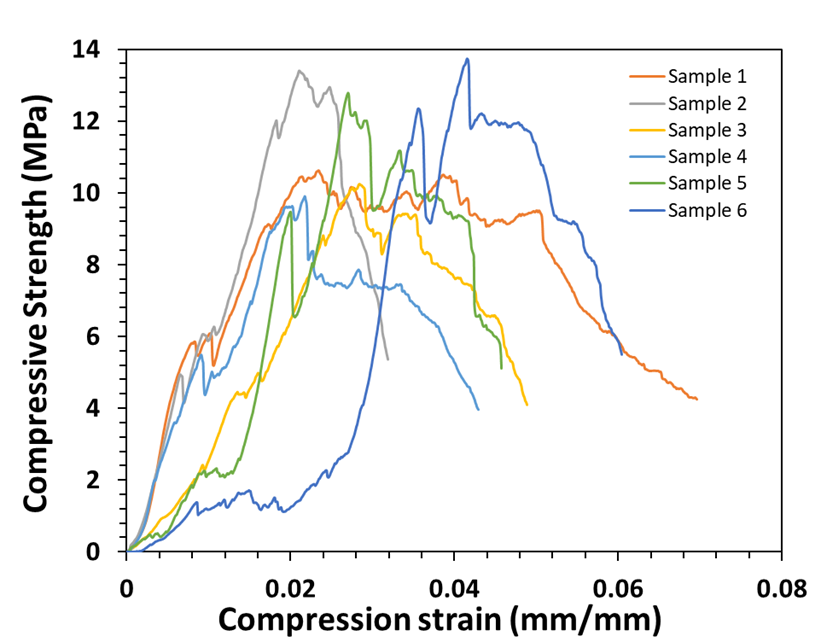


**sFig. 2** A universal testing machine determined stress-strain curves of compression of sintered 3D-printed bioceramic tested specimens.

In the process of preparing tissue samples for immunohistochemistry of type II collagen, the tissue was carefully neutralized using a 5% sodium sulfate solution and allowed to incubate for a period of 2 hours. This step is vital in preventing any undesired chemical reactions within the tissue. Following this, the tissue is diligently rinsed with running water for a duration of 30 minutes, ensuring that all residual substances are thoroughly removed.

Next, involves the removal of endogenous peroxidase using H_2_O_2_, a process that takes just 10 minutes. Then, antigen retrieval is carried out using a Citrate buffer with a pH of 6.0, executed within a pressure cooker for a span of 1 hour. This step aims to unmask the target antigens, making them accessible for the antibody to bind.

The tissue is treated with Collagen II at a 1000× dilution, and this incubation is performed at room temperature overnight to facilitate specific antibody binding. Introduce Goat Probe HRP labeling for 15 minutes and Rabbit HRP for 30 minutes, respectively, enhancing the visualization of the target molecules within the tissue.The final step involves the application of DAB (3,3'-Diaminobenzidine) for 20 minutes. This substrate, when exposed to peroxidase enzymes, develops into a brown precipitate, marking the presence of the target antigen.

**sFig. 3** Type Ⅱ collagen analyses of repaired Osteochondral tissue.

**sFig. 4** Soft X-ray photographs of osteochondral defect of femoral trochlear in other typic rabbit cartilage experimental groups. Osteochondral defects immediately after the surgery (a) Osteochondral repair defect before sacrificing at 8 weeks (b).

**
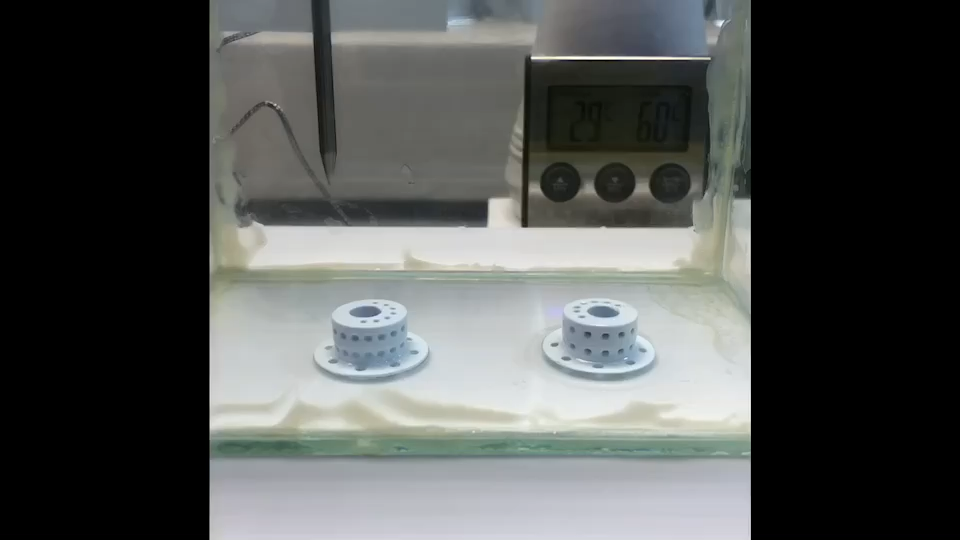
**

**Video.** The negative temperature-responsive (NTR) bioceramic characteristics exhibit drainage shrinkage effects over a temperature range of 29°C to 60°C. Without silicon oil enveloped (left sample) and with silicon oil enveloped (right sample).
